# Arginine-modified black phosphorus quantum dots with dual excited states for enhanced electrochemiluminescence in bioanalysis

**DOI:** 10.1038/s41467-022-35015-9

**Published:** 2022-11-26

**Authors:** Siqi Yu, Yu Du, Xianghong Niu, Guangming Li, Da Zhu, Qian Yu, Guizheng Zou, Huangxian Ju

**Affiliations:** 1 State Key Laboratory of Analytical Chemistry for Life Science, School of Chemistry and Chemical Engineering, Nanjing University, Nanjing, 210023 P. R. China; 2 School of Science, Nanjing University of Posts and Telecommunications, Nanjing, 210023 P. R. China; 3 School of Chemistry and Chemical Engineering, Shandong University, Jinan, 250100 P. R. China

**Keywords:** Bioanalytical chemistry, Quantum dots, Materials for optics

## Abstract

The electrochemiluminescence (ECL) is generally emitted via radiative transition of singlet or triplet excited state (S_1_ or T_1_). Herein, an ECL mechanism with the transitions of both S_1_ and T_1_ of black phosphorus quantum dots (BPQDs) is found, and an arginine (Arg) modification strategy is proposed to passivate the surface oxidation defects of BPQDs, which could modulate the excited states for enhancing the ECL efficiency of BPQDs. The Arg modification leads to greater spatial overlap of highest and lowest occupied molecular orbitals, and spectral shift of radiative transitions, and improves the stability of anion radical of BPQDs. To verify the application of the proposed mechanism, it is used to construct a sensitive method for conveniently evaluating the inhibiting efficiency of cyclo-arginine-glycine-aspartic acid-d-tyrosine-lysine to cell surface integrin by using Arg containing peptide modified BPQDs as signal tag. The dual excited states mediated ECL emitters provide a paradigm for adjustable ECL generation and extend the application of ECL analysis.

## Abstract (web summary)

Electrochemiluminescence is emitted via the radiative transition of a singlet or triplet excited state. Here, the authors propose an arginine modification of black phosphorus quantum dots that exhibits enhanced emission based on dual excited states.

## Introduction

Electrochemiluminescence (ECL) is a light-emitting process, in which the excited state species (R^*^) are generated via exergonic electron transfer and exchange between the electrogenerated intermediates, following the radiative transitions to the ground state (S_0_)^1^. Generally, the produced R^*^ can be either the lowest excited singlet state (S_1_) species (^1^R^*^) or the triplet state (T_1_) species (^3^R^*^), depending on the difference of their relative energy^2–5^. According to the rules of spin statistics, the internal quantum efficiency of the luminophores emitting ECL is limited to 25% due to their ^1^R^*^-to-^3^R^*^ ratio of 1:3 and the non-radiative transition from T_1_ to S_0_^6^, which limits the full utilization of the energy during the ECL process. In order to harvest both singlet and triplet excitons for electroluminescence emission, thermally activated delayed fluorescence (TADF) emitters have been designed to achieve a high luminescent efficiency via rapid reverse intersystem crossing (RISC) of T_1_ excitons to S_1_ excitons^7^. The TADF usually occurs only when the energy gap between S_1_ and T_1_ (Δ*E*_*ST*_) is sufficiently small (ca. 0.1 eV)^8^, and has been used for the development of organic light-emitting diodes^7,9^ and aqueous TADF-ECL systems^10^. However, the ECL behaviors of TADF emitters are mainly studied in organic media^6^, and the ECL efficiency of 0.7% vs. Ru(bpy)_3_^2+^ for aqueous TADF-ECL system is relatively low^11^, which greatly limits their applications in biosensing and bioanalysis. In this work, we found a possibility to emit the ECL via both S_1_-to S_0_ and T_1_-to-S_0_ transitions by using black phosphorus quantum dots (BPQDs) as the emitter.

BPQDs were firstly prepared in 2014 with bulk black phosphorus (BP)^12,13^, which is a metal-free semiconductor and displays tunable bandgap varying from 0.3 eV for bulk BP to 2.0 eV for monolayer BP^14^, and have been extensively applied in photothermal therapy, electrocatalysis and flexible devices^12,13,15–17^. Although the ECL emissions of BPQDs^18,19^ and BP nanosheets^20–22^ have been observed, the optical and electrical performances of BP nanomaterials are greatly limited by the oxidation defects of nanostructure surface due to the easy degradability under ambient conditions^23–25^. Therefore, great efforts have been made to stabilize BPQDs by either preventing the occurrence of oxidation process via encapsulation with polyethylene glycol, fluorine, and poly(lactic-co-glycolic acid)^14,16,26^, or passivating the existing oxidation defects with ethanol^27^. Meanwhile, arginine (R or Arg) contained peptides and poly-l-lysine have also been used to modify BP nanosheets via electrostatic and/or hydrophobic interaction for the preparation of delivery carrier and the immobilization of protein, respectively^28,29^. Inspired by these interaction mechanisms, this work designed a strategy to passivate the oxidation defects of BPQDs with Arg for enhancing the ECL performance.

Different from early reported cathodic ECL emissions of BPQDs centering at 555 and 640 nm, which were assigned to the emissions from the bandgap and the surface states^18^, here the ECL emissions of BPQDs prepared with another procedure occur at 500 and 580 nm, and can be attributed to the direct radiative transitions of both ^1^R^*^ and ^3^R^*^ to S_0_ species, respectively. The latter is generally observed in the ECL emission of some organic small molecules with phosphorescence (PL) properties, such as benzophenone, platinum-based organometallics, and tris(1-phenyl isoqsuinoline-C2, N)iridium (III)^30–32^. Interestingly, the cathodic and anodic ECL emission upon Arg modification of BPQDs increased dramatically by 25 and 2 folds due to the passivation of Arg to surface oxidation defects, respectively, which resulted in the change of the highest occupied molecular orbital (HOMO) from the surface oxidation defects to the central zone of arginine modified BPQDs (R-BPQDs), and thus greater spatial overlap of the HOMO with the lowest unoccupied molecular orbital (LUMO), as proved by time-dependent density functional theory (TD-DFT) calculations, and the adjustable transition routes from S_1_ and T_1_ to S_0_. Thus Arg modification could be used to efficiently modulate the excited states for enhancing the ECL emission. Due to the improved stability of anion radical of BPQDs, the cathodic ECL showed greater improvement of emission performance than anodic ECL. More importantly, the Arg functionalization improved the solubility and modifiability of BPQDs for extending the bioanalytical applications. As an example, a cathodic ECL system was proposed for conveniently evaluating the integrin inhibitor by using arginine-arginine-glycine-aspartic acid-serine (RRGDS) peptide-modified BPQDs (RRGDS-BPQDs) as signal tag to recognize cell surface integrin. This work demonstrated a dual-excited-state ECL mechanism and an avenue to modulate the excited states for enhancing the ECL emission of luminophores.

## Results

### Characterization of BPQDs and R-BPQDs

**Fig. 1:**
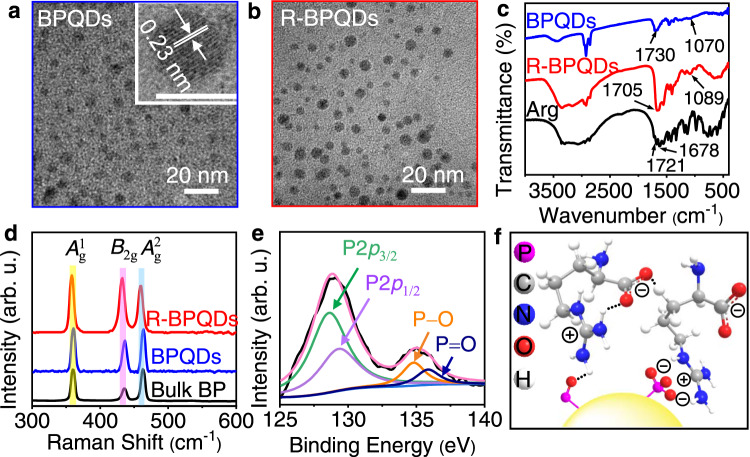
Structural and chemical characterization before (BPQDs, blue) and after Arg modification (R-BPQDs, red). **a**, **b** TEM images. Inset in (**a**) HRTEM image of BPQDs; scale bar: 5 nm. **c** FTIR spectra with Arg as reference (black). **d** Raman spectra with bulk BP as reference (black). **e** Experimental XPS data (black) of R-BPQDs and fitted spectrum (pink; including P2*p*_3/2_ in green, P2*p*_1/2_ in purple, P–O in orange, *P* = O in dark blue). **f** Schematic profile of interaction between BPQDs and Arg. Source data are provided as a Source Data file.

From the transmission electron microscopic (TEM) images, the average lateral sizes of BPQDs and R-BPQDs were measured to be about 4 nm (Fig. 1a, b). The lattice fringe of 0.23 nm could be ascribed to the (041) plane of the BP crystal (Inset in Fig. 1a)^33^. The measured heights of 1.1, 1.8, and 2.4 nm (Supplementary Fig. 1) corresponded to BPQDs with about 2-4 layers, respectively^12,29^. The zeta potential of BPQDs in an aqueous solution was −26.2 mV due to the surface oxidation defects^13,34^, which was changed to −67.8 mV after Arg modification (Supplementary Fig. 2), indicating the electrostatic interaction of the surface defects with a delocalized positive charge of guanidine group to expose the carboxyl group of Arg with a pKa value of 2.17^35^.

The Fourier transform infrared (FTIR) spectra showed the shift of P − O bending peak from 1070 cm^−1^ of BPQDs to 1089 cm^−1^ of R-BPQDs (Fig. 1c)^36,37^, and that the N − H bending vibration peak in guanidine group at 1721 cm^−1^, as a shoulder peak of COO^−^ asymmetric stretching at 1678 cm^−1^, experienced a red-shift to 1705 cm^−1^ in R-BPQDs as a result of its coexistence with the peaks at 1678 cm^−1^ for COO^−^ asymmetric stretching of Arg and 1730 cm^−1^ for P = O vibration peak of BPQDs. Besides, the peaks of BPQDs at 2895 and 2973 cm^−1^ were assigned as P − O vibration and 3473 cm^−1^ as O − H vibration of BPQDs and H_2_O, while the peaks of Arg from 2895 to 3115 cm^−1^ were associated with C − H stretching vibration, and the peaks from 3346 to 3410 cm^−1^ were related to N − H stretching vibration (Supplementary Fig. 3 and Supplementary Table 1)^38,39^. The FTIR spectrum of R-BPQDs showed the integrated results of Arg and BPQDs from about 2700 to 3500 cm^−1^. These results indicated the electrostatic and hydrogen bond interactions between the electron-withdrawing guanidine group in Arg and P_x_O_y_ moiety as previous report^34,40^. The Arg modification also led to slight red-shifts of three prominent Raman peaks of BPQDs (Fig. 1d), identified as one out-of-plane phonon mode (*A*^1^_g_) at 360.1 cm^−1^ and two in-plane modes (*B*_2g_ and *A*^2^_g_) at 435.5 and 462.7 cm^−1^ ^12,13^, which manifested a change of the Raman scattering wavelength due to the interaction between guanidine group and P_x_O_y_ moiety^41^. Moreover, these Raman peaks were relatively stable upon storage, while the Raman peaks of BPQDs almost vanished after 30 day-storage^25^ (Supplementary Fig. 4), verifying the better storage stability of R-BPQDs.

**Table 1:** ECL responses of GCEs modified with 20 kinds of amino acid-functionalized BPQDs in 0.1 M pH 7.4 PBS containing 0.1 M K_2_S_2_O_8_ and pIs of corresponding amino acids

Amino acid-functionalized BPQDs	Peak potential (V vs. Ag/AgCl)	Maximum emission (arb. u.)	pI of amino acid^35^
G-BPQDs	−1.60 V	309	5.97
A-BPQDs	−1.55 V	377	6.00
V-BPQDs	−1.45 V	612	5.96
L-BPQDs	−1.33 V	513	5.98
I-BPQDs	−1.42 V	631	6.02
M-BPQDs	−1.35 V	293	5.74
F-BPQDs	−1.60 V	398	5.48
W-BPQDs	−1.60 V	340	5.89
P-BPQDs	−1.36 V	520	6.30
S-BPQDs	−1.60 V	850	5.68
T-BPQDs	−1.60 V	896	6.16
C-BPQDs	−1.60 V	456	5.05
Y-BPQDs	−1.60 V	687	5.66
N-BPQDs	−1.60 V	746	5.41
Q-BPQDs	−1.60 V	442	5.64
D-BPQDs	−1.36 V	501	2.77
E-BPQDs	−1.60 V	486	3.22
K-BPQDs	−1.60 V	947	9.74
R-BPQDs	−1.20 V	1982	10.8
H-BPQDs	−1.60 V	316	7.59

*G* glycine, *A* alanine, *V* valine, *L* leucine, *I* isoleucin, *M* methionine, *F* phenylalanine, *W* tryptophan, *P* proline, *S* serine, *T* threonine, *C* cysteine, *Y* tyrosine, *N* asparagine, *Q* glutamine, *D* aspartic acid, *E* glutamic acid, *K* lysine, *H* histidine.

The R-BPQDs retained the characteristic P2*p*_3/2_ and P2*p*_1/2_ X-ray photoelectron spectroscopic (XPS) peaks of BP at 128.6 and 129.3 eV (Fig. 1e and Supplementary Fig. 5a, b), while BPQDs did not show these peaks, demonstrating that the introduction of Arg endowed BPQDs with better stability. The shifts of P − O and P = O XPS peaks from 132.6 and 133.6 eV of BP to 134.8 and 135.9 eV of R-BPQDs could be attributed to the decrease of the outer valence electron density of P atom in the presence of guanidine group (Fig. 1e and Supplementary Fig. 5b), which did not obviously change the N1*s* XPS peak due to the delocalized positive charge distribution over the guanidine group (Supplementary Fig. 5c, d), but increased the binding energy of O atom for P−O and P=O bonds due to the decreased outer valence electron density (Supplementary Fig. 5e–h). In a word, the presence of Arg on the PxOy moiety of BPQDs exerted a blocking effect against the oxidation defects of BPQDs via electrostatic and hydrogen bond interactions (Fig. 1f), and endowed BPQDs with better stability under ambient conditions.

### Photophysical and electrochemical properties of BPQDs and R-BPQDs

**Fig. 2:**
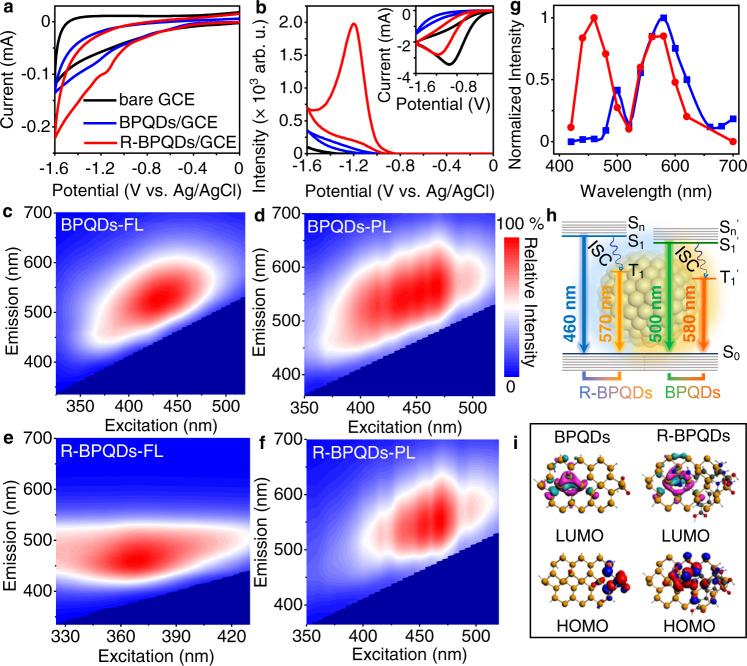
Cathodic ECL and photophysical properties before (BPQDs/GCE, blue) and after Arg modification (R-BPQDs/GCE, red). **a** CVs of glassy carbon electrodes (GCEs) in 0.1 M PBS. **b** ECL curves of GCEs in 0.1 M pH 7.4 PBS containing 0.1 M K_2_S_2_O_8_. PMT = 600 V. Inset: CVs for (**b**). **c**–**f** Excitation-emission maps for fluorescence (FL; **c**, **e**) and phosphorescence (PL; **d**, **f**) before (**c**, **d**) and after Arg modification (**e**, **f**). **g** Normalized cathodic ECL spectra of BPQDs/GCE (blue) and R-BPQDs/GCE (red). **h** Schematic illustration of ECL transitions. **i** Isosurfaces of HOMO and LUMO wavefunctions. Source data are provided as a Source Data file.

The cathodic electrochemical process of BPQDs/GCE showed a weak reduction peak of BPQDs at around −1.22 V, which shifted to −1.15 V and became more distinct after introducing the electron-withdrawing guanidine group by Arg modification (Fig. 2a). In the presence of K_2_S_2_O_8_, R-BPQDs/GCE showed a cathodic ECL emission at −1.20 V, which was 25 folds stronger than that of BPQDs/GCE (Fig. 2b). The cathodic ECL efficiency of R-BPQDs was 3.2 times higher compared to BPQDs. The relative cathodic ECL efficiency of R-BPQDs and BPQDs was 48% and 15% vs. 1 mM [Ru(bpy)_3_]^2+^ with 0.1 M K_2_S_2_O_8_ as co-reactant, respectively.

The ECL emission followed a general K_2_S_2_O_8_ mediated co-reactant ECL mechanism containing the electro-reduction of K_2_S_2_O_8_ and BPQDs or R-BPQDs (Inset in Fig. 2b) and the oxidation of the formed BPQDs^·^^−^ or R-BPQDs^·−^ by the produced SO_4_^·−^ to produce the excited state BPQDs^*^ or R-BPQDs^*^^4,42^. The reduction peak of K_2_S_2_O_8_ at bare GCE occurred at −0.95 V, and almost disappeared at BPQDs/GCE due to the greatly increased electron transfer impedance (*Re*) (Supplementary Fig. 6). The much lower *Re* than BPQDs/GCE led to obvious reduction peak of K_2_S_2_O_8_ at R-BPQDs/GCE, which overlapped with the reduction peak of R-BPQDs (Fig. 2a). The consistence of onset potentials for electrochemical reduction and cathodic ECL emission of R-BPQDs indicated that the electrogeneration of R-BPQDs^•−^ was necessary for the formation of excited state R-BPQDs^*^^43^. Interestingly, the ECL emission depended on both Arg concentration and pH for the preparation of R-BPQDs (Supplementary Fig. 7), implying that the amount of Arg assembled on BPQDs affected the ECL performance, though Arg did not participate in the ECL process (Supplementary Fig. 8).

The excitation-emission matrix spectra of BPQDs showed the FL and PL emission centering at 520 and 565 nm, respectively (Fig. 2c, d), which originated from the oxidation defects associated S_1_ and T_1_, respectively, and could be demonstrated by the photoluminescence decay spectrum (PDS) (Supplementary Fig. 9). The PDS of BPQDs gave the average lifetimes of 11.86 μs for PL emission and 10.66 ns for FL emission (Supplementary Tables 2, 3)^44^. Compared to BPQDs, R-BPQDs displayed 60-nm and 5-nm blue shifts of FL and PL emissions, respectively (Fig. 2e, f). The hypochromic shift of FL emission was probably related to the change of the excited states due to the passivation of oxidation defects by Arg. Similarly, the PDS of FL and PL emissions of R-BPQDs gave the average decay lifetimes of 9.40 ns and 12.95 μs (Supplementary Tables 2 and 3). Excitingly, the cathodic ECL spectra of both BPQDs and R-BPQDs displayed two emission peaks at 500 and 580 nm for BPQDs, and 460 and 570 nm for R-BPQDs (Fig. 2g), which almost located at the same positions with the FL and PL emission peaks, and could be assigned to the two radiative transitions: S_1_-to-S_0_ and T_1_-to-S_0_ emission, as shown in Fig. 2h. The existence of two excited states of both R-BPQDs and BPQDs was also proved by the UV-vis-NIR diffuse reflectance spectra (Supplementary Fig. 10), which showed two experimental band gaps calculated from the Tauc plots, in accordance with their FL and PL excitations, respectively. Besides, the lifetimes did not obviously change at temperatures from 170 to 310 K (Supplementary Fig. 11 and Table 4), so the possibility of TADF process could be excluded^6,45^, further indicating the existence of two radiative transitions from both S_1_ and T_1_ for ECL emission.

According to the electrostatic and hydrogen bond interactions between Arg and oxidation defects of BPQDs, TD-DFT computation was implemented to rationalize the above conclusion. The existence of oxidation defects resulted in the localized HOMO of BPQDs at the defect sites (Fig. 2i), which hindered the charge transfer as a trap state, and thus weakened the emission intensity. The Arg modification passivated the surface oxidation defects, accordingly leading to the delocalization of HOMO of R-BPQDs to the central zone (Fig. 2i), and thus the change of the electron transition channel, which significantly improved the emission oscillator strength and the charge transfer capability (Supplementary Fig. 6).

The FL quantum yield of BPQDs increased from 1.99% to 18.63% after Arg modification, further confirming that the change of HOMO from the surface oxidation defects to the central zone of R-BPQDs could facilitate S_1_-to-S_0_ radiative transition. To evaluate the significance of Arg, the ECL performances of 20 kinds of amino acid-functionalized BPQDs prepared with the same method as R-BPQDs were compared in Table 1. It was concluded that R-BPQDs exhibited the strongest ECL emission and the most positive reduction potential due to its highest isoelectric point (pI)^35^ and the presence of electron-withdrawing guanidine group, which stabilized the adjacent R-BPQDs^•−^ anion radical after electrochemically injecting electron into LUMO of R-BPQDs^46,47^, and thus facilitated the cathodic ECL emission.

**Fig. 3:**
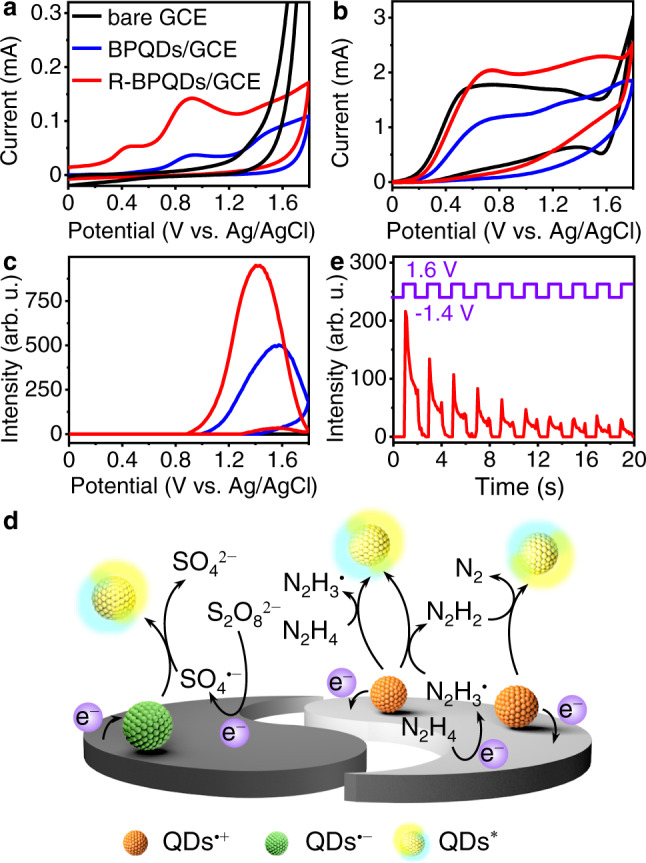
Anodic ECL and transient ECL before (BPQDs/GCE, blue) and after Arg modification (R-BPQDs/GCE, red). **a**, **b** CVs and **c** anodic ECL curves with bare GCE as reference (black) in 0.1 M pH 7.4 PBS in the absence (**a**) and (**b**, **c**) presence of 25 mM N_2_H_4_∙H_2_O. PMT = 600 V. **d** Proposed mechanism of cathodic and anodic ECL for Arg-modified BPQDs/GCE in the presence of K_2_S_2_O_8_ or N_2_H_4_∙H_2_O, respectively. **e** Transient ECL of R-BPQDs/GCE in 0.1 M PBS by stepping potential (purple) from −1.4 V to +1.6 V at 1 Hz. Source data are provided as a Source Data file.

In the anodic process, the BPQDs/GCE showed two anodic peaks at +0.85 and +1.45 V (Fig. 3a), which were attributed to the electrochemical oxidation of surface groups such as phosphite and hypophosphoric groups^34,48^. These peaks negatively shifted to +0.42 and +0.86 V after Arg modification (Fig. 3a) due to the much lower *Re* (Supplementary Fig. 6), which decreased the oxidation overpotentials. Although the oxidation of Arg could be observed at bare GCE at +1.62 V (Supplementary Fig. 12), it did not occur at R-BPQDs/GCE in the applied potential range due to the relative higher *Re*. Considering the low oxidation potential of R-BPQDs, N_2_H_4_∙H_2_O that can be electrochemically oxidized to produce N_2_H_3_^•^ and N_2_H_2_ around +0.1 V was used as co-reactant to study the anodic ECL of BPQDs and R-BPQDs^49,50^. At bare GCE, the oxidation of N_2_H_4_∙H_2_O occurred near +0.10 V, which showed a peak at +0.55 V and a severe tailed anodic curve due to the further oxidation of N_2_H_3_^•^ and N_2_H_2_ at higher potentials (Fig. 3b). Obviously, the anodic curve of N_2_H_4_∙H_2_O positively shifted due to the increased *Re*, and covered the oxidation peaks of BPQDs at both BPQDs/GCE and R-BPQDs/GCE. Furthermore, the hole-injected BPQDs (BPQDs^•+^) could oxidize the reducing N_2_H_3_^•^, N_2_H_2,_ and N_2_H_4_ to form the excited state species BPQDs^*^ or R-BPQDs^*^ for ECL emission (Fig. 3c). The anodic ECL peak potential and intensity of R-BPQDs were 0.15 V lower and 2 times higher than those of BPQD. The anodic ECL efficiency of R-BPQDs was 1.2 times higher compared to BPQDs, and the relative anodic ECL efficiency of R-BPQDs and BPQDs was 0.21% and 0.18% vs. 1 mM [Ru(bpy)_3_]^2+^ with 10 mM TPA as co-reactant, respectively. The higher ECL efficiency of R-BPQDs could be attributed to the better charge transfer capability, the greater spatial overlap between HOMO and LUMO, and the better stability under ambient conditions after Arg modification. Similar to the cathodic ECL process, the anodic ECL spectra of BPQDs and R-BPQDs also displayed two emission peaks associating S_1_ and T_1_ transitions, along with the hypochromic shifts (Supplementary Fig. 13). Thus, the co-reactant ECL mechanisms of R-BPQDs at the cathode and the anode could be illustrated in Fig. 3d.

ECL transient technology was further used to examine the stability of radical intermediates in two ECL processes of R-BPQDs. The ion annihilation ECL intensity at +1.60 V was stronger than that at −1.40 V in the absence of coreactant (Fig. 3e), indicating that the anion radical R-BPQDs^•−^ was more stable than cation radical R-BPQDs^•+^^51^. Thus Arg stabilized the anion radical of BPQDs under ambient conditions, and thus led to the greater enhancement of cathodic ECL intensity than the anodic process.

### Evaluation of integrin inhibitor with RRGDS-BPQDs

**Fig. 4:**
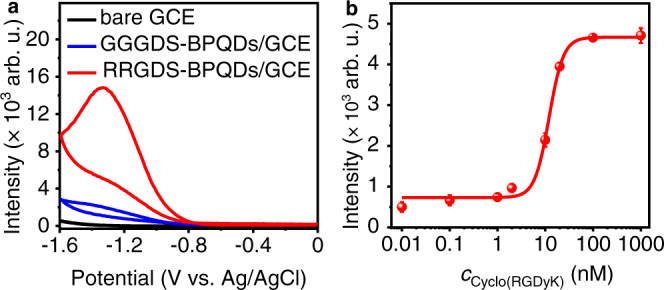
Evaluation of integrin inhibitor cyclo(RGDyK). **a** ECL curves with Arg-free peptide (GGGDS-BPQDs/GCE, blue) and Arg-containing peptide (RRGDS-BPQDs/GCE, red) in 0.1 M PBS containing 0.1 M K_2_S_2_O_8_ with bare GCE as reference (black). PMT = 800 V. **b** ECL response of RRGDS-BPQDs/MWNTs/GCE to 1 × 10^6^ A549 cells mL^−1^ pretreated with 0.01, 0.1, 1, 2, 10, 20, 100, and 1000 nM cyclo(RGDyK). The error bars represent the SD from 3 measurements. Data are expressed as means ± SD. Source data are provided as a Source Data file.

To implement the ECL application of R-BPQDs, this work used R-BPQDs/K_2_S_2_O_8_ system to design an ECL method for evaluating the inhibition efficiency of integrin inhibitor, cyclo-arginine-glycine-aspartic acid-d-tyrosine-lysine (cyclo(RGDyK))^52^. The BPQDs were firstly modified with Arg-containing peptide RRGDS to functionalize carboxylated multi-wall carbon nanotubes (MWNTs), which were then coated on GCE to act as both the recognition unit and signal tag^53^. Compared to Arg-free glycine-glycine-glycine-aspartic acid-serine (GGGDS) peptide, the presence of RRGDS peptide could greatly enhance the ECL intensity (Fig. 4a), verifying the vital importance of Arg for improving the ECL emission of BPQDs. Upon the specific recognition of αV/β3 integrin on A549 cell membrane with RRGDS, A549 cells were effectively captured onto the electrode surface (Supplementary Fig. 14), which led to the greatly increased *Re* (Supplementary Fig. 15), and thus the obviously decreased ECL intensity (Supplementary Fig. 16). In contrary, MCF-7 cells with low abundance of surface αV/β3 integrin showed little decrease. Under optimal conditions (Supplementary Figs. 17 and 18), the IC50 of cyclo(RGDyK) for 1 × 10^6^ A549 cells mL^−1^ was obtained to be 12.0 nM from the ECL response plot of cyclo(RGDyK) treated A549 cells (Fig. 4b), which was comparable to 20 nM for immobilized αV/β3 integrin^52^ and 56.2 nM for U87MG cells^54^. The ECL intensity of A549/RRGDS-BPQDs/MWNTs/GCEs during continuous three cycles of electrochemical scanning at different concentrations of cyclo(RGDyK) demonstrated good measurement stability (Supplementary Fig. 19). Ten A549/RRGDS-BPQDs/MWNTs/GCEs with a relative standard deviation (RSD) of 2.7% (Supplementary Fig. 19) showed favorable electrode-to-electrode reproducibility of the ECL sensor. Compared to general methods for IC50 determination of cyclo(RGDyK) or its analogues, such as (3-(4,5-imethylthiazol-2-yl)−2,5-diphenyltetrazolium bromide-based spectrophotometry^55^ and radioimmunoassay^56^, this method possessed the shortest analytical time (Table 2). Moreover, this method could be extended for the evaluation of other inhibitors or the detection of cell surface groups by changing the Arg-containing peptide, showing the excellent practicability of the designed ECL modulating strategy.

**Table 2:** Analytical performance of biosensors for IC50 determination of cyclo(RGDyK) or its analogues

Method	Indicator	Biocompatibility	Assay time	Reference
MTT assay	Formazan	Bad	51 h	^55^
CCK-8 assay	Formazan	Bad	25 h	^62^
ELISA	TMB	Good	4 h	^63^
Radioimmunoassay	^125^I-labeled echistatin	Bad	3 h	^56^
Chemiluminescence	Galacton-Plus	Bad	195 min	^64^
Fluorescence	Alamar blue	Good	72 h	^65^
ECL	RRGDS-BPQDs	Good^16^	170 min	This work

*MTT* (3-(4,5-imethylthiazol-2-yl)−2,5-diphenyltetrazolium bromide, *CCK-8* cell counting kit-8, *ELISA* enzyme linked immune sorbent assay, *TMB* 3,3’,5,5’-tetramethylbenzidine.

## Discussion

The BPQDs can be conveniently modified with Arg via the electrostatic and the hydrogen bond interaction between the electron-withdrawing guanidine group and P_x_O_y_ moiety. The presence of Arg on BPQDs passivates the oxidation defects of BPQDs and increases the negative surface charge due to the exposure of carboxyl group with a pKa value of 2.17, and thus endows BPQDs with better stability, solubility and modifiability for extending the bioanalysis application.

The ECL emission of BPQDs shows a mechanism containing two radiative transitions from both S_1_ and T_1_ to S_0_, which has been demonstrated by the ECL spectra with two emission peaks as observed from the FL and PL emission of BPQDs, and the PDS showing both ns- and μs-level decay lifetimes, and also proved by two experimental band gaps calculated from the Tauc plots of the UV-vis-NIR diffuse reflectance spectra. Thus a dual excited states mediated ECL emitter has been found.

The Arg modification of BPQDs greatly enhances the ECL intensity, which is attributed to the passivation of the surface oxidation defects to produce the delocalization of HOMO of R-BPQDs to the central zone. The passivation changes the electron transition channel and has been demonstrated by TD-DFT computation. By comparing the ECL changes upon different amino acid modifications, it has been concluded that the cathodic ECL enhancement of R-BPQDs is attributed to the presence of electron-withdrawing guanidine group, which stabilizes the adjacent R-BPQDs^•−^ anion radical after electrochemically injecting electron into LUMO of R-BPQDs. Thus the relative cathodic ECL efficiency of R-BPQDs vs. 1 mM [Ru(bpy)_3_]^2+^ with 0.1 M K_2_S_2_O_8_ can reach 48%, which is much larger than those of TADF emitters reported previously, providing an alternative ECL nanoemitter for bioanalysis.

To demonstrate the application of R-BPQDs in ECL bioanalysis, the Arg attached on BPQDs has been replaced by Arg-containing peptide RRGDS, where RGDS is a peptide specific to integrin. By coating RRGDS-BPQDs on MWNTs modified GCE, the integrin-rich cells can be bound to the electrode surface via the recognition of RGDS to integrin^57^, which leads to a sensitive ECL method for the evaluation of integrin inhibitor. The obtained inhibiting efficiency to integrin on A549 cells has demonstrated the practicability of the ECL of BPQDs and the modulating strategy.

In summary, both S_1_-to-S_0_ and T_1_-to-S_0_ radiative transitions have been found in both cathodic and anodic ECL emissions of BPQDs, different from the early reported bandgap luminescence and the surface state luminescence^18^. Arg modification efficiently passivates the oxidation defects of BPQDs and changes the HOMO from surface defects to the central zone, thus leading to hypochromic shifts and intensity enhancement of ECL emission. The introduction of Arg changes the electron transition channel and endows BPQDs with better stability, stronger charge transfer capability, and greater spatial overlap between HOMO and LUMO. The presence of electron-withdrawing guanidine group greatly stabilizes the anion radical intermediates, and thus leads to greater enhancement of the cathodic ECL efficiency. The proposed modulating strategy can be conveniently applied in biosensing by using different Arg-containing recognition units to modify BPQDs, which has been demonstrated by using RRGDS-BPQDs to evaluate the inhibiting efficiency of cyclo(RGDyK) to cell surface integrin. Nonetheless, alternative explanations for the bi-exponential decays in the luminescence transients exist as for example, two populations of QDs or two differing chemical environments. Thus, further experimental work will be necessary to give final proof of the proposed mechanisms of dual excited states.

## Methods

### Materials and reagents

N-hydroxysuccinimide (NHS, 98%), l-arginine (R, ≥98%), l-histidine (H, ≥99%), 1-(3-(dimethylamino)propyl)−3-ethylcarbodiimide hydrochloride (EDC, 99%) and hydrazine hydrate solution (N_2_H_4_·H_2_O, 64-65%) were purchased from Sigma-Aldrich (Merck, USA). 1-Methyl-2-pyrrolidinone (NMP, 99.5%) was purchased from J&K Chemical Technology Co., Ltd. (Beijing, China). l-Lysine monohydrochloride (K, 99%), L-isoleucine (I, ≥98.5%), l-valine (V, 98%), l-asparagine monohydrate (D, ≥98.5%), l-phenylalanine (F, 99%), l-leucine (L, 99%), l-threonine (T, 99%), l-glutamine (Q, ≥99%), l-alanine (A, ≥98.5%), l-serine (S, 95%), l-glutamic acid (E, ≥98.5%), potassium hexacyanoferrate(III) (K_3_[Fe(CN)_6_], ≥99.5%), potassium hexacyanoferrate(II) trihydrate (K_4_[Fe(CN)_6_·3H_2_O], ≥99.5%) and potassium peroxydisulfate (K_2_S_2_O_8_, ≥99.5%) were purchased from Hushi Co., Ltd. (Shanghai, China). l-Aspartic acid (D, ≥99%), l-glycine (G, ≥98.5%), l-proline (P, ≥98.5%), l-cysteine (C, ≥98.5%), l-methionine (M, 99%), l-tryptophan (W, 98%) and l-tyrosine (Y, 99%) were obtained from Ryon Biological Technology Co., Ltd. (Shanghai, China). Bulk back phosphorus (BP, >99.998%) and carboxylated multi-walled carbon nanotubes (MWNTs) (>95%, 20-30 nm diameter, 0.5-2 µm length) were obtained from Jiangsu XFNANO Materials Tech Co., Ltd. (Nanjing, China). 2-(N-morpholino)ethanesulfonic acid (MES) buffer (0.1 M, pH 6.0) was purchased from Coolaber Technology Co., Ltd. (Beijing, China). Peptides, including RRGDS (98.945%) and GGGDS (99.899%), were obtained from Sangon Biotech Co., Ltd. (Shanghai, China). The inhibitor of αV/β3 integrin, cyclo(RGDyK) (99.85%), was purchased from topscience Co., Ltd. (Shanghai, China). Fetal bovine serum (FBS) was obtained from Thermo Fisher Scientific Inc. (USA). A549 cells, MCF-7 cells, trypsin and cell culture media (RPMI-1640) were supplied by KeyGen Biotech Co., Ltd. (Nanjing, China). All chemicals were of analytical grade. Phosphate buffer solution (PBS, 0.1 M, pH 7.4) was prepared by mixing stock solutions of NaH_2_PO_4_ and Na_2_HPO_4_. ECL measurements were conducted in 0.1 M pH 7.4 PBS containing 0.1 M KNO_3_ or 0.1 M KCl as the electrolyte. All aqueous solutions were prepared using ultrapure water (≥ 18 MΩ·cm, Milli-Q, Millipore).

### Preparation and functionalization of BPQDs

The BPQDs were prepared by top-down solvothermal treatment according to the literature with some modification^12^. Briefly, 15 mg of bulk BP crystals was firstly grounded into powder by a mortar and pestle in the glovebox, which was then dispersed in 15 mL of anhydrous NMP to heat for 12 h at 140 °C under Ar atmosphere. The resulting dark brown dispersion was centrifuged at 7300 *g* for 20 min to remove the oversized particles. The supernatant was collected and further centrifuged at 36,900 *g* for 40 min. The resultant BPQDs were redispersed in 15 mL of anhydrous NMP and kept in refrigerator at 4 °C for further use. To prepare amino acid- or peptide-functionalized BPQDs, 1.0 mL of BPQDs dispersion was centrifuged at 50,300 *g* for 40 min. The resultant BPQDs were redispersed in 1.0 mL of amino acid or peptide solution and sonicated in an ice-bath for 30 min. Afterward, the mixture was shaken overnight at room temperature away from light. The resultant solution was centrifuged at 50,300 *g* for 40 min and washed three times with water. Finally, the amino acid-functionalized BPQDs, RRGDS-BPQDs, and GGGDS-BPQDs were redispersed in deoxygenated water and kept at 4 °C in the dark.

### Cell culture and inhibitor treatment

A549 and MCF-7 cells were cultured in RPMI-1640 media supplemented with 10% FBS and 100 µg mL^−1^ penicillin-streptomycin at 37 °C in a humidified atmosphere containing 5% CO_2_ to maintain a density between 5 × 10^5^ and 2 × 10^6^ cells mL^−1^. At the logarithmic growth phase, the cells were trypsinized and washed twice with sterile 0.01 M pH 7.4 PBS by centrifugation at 100 *g* for 10 min, which were then resuspended in 0.01 M pH 7.4 PBS containing 1.0 mM Ca^2+^ and 1.0 mM Mg^2+^ to obtain a homogeneous cell suspension. The inhibitor treatment of the cells was performed by incubating 200 μL of 1 × 10^6^ A549 cells mL^−1^ for 20 min with different concentrations of αV/β3 integrin inhibitor, cyclo(RGDyK), on a rotary shaker in the dark at 37 °C. After washing with PBS for three times, the treated cells were redispersed in 200 μL of 0.01 M pH 7.4 PBS for ECL evaluation of inhibiting efficiency.

### Fabrication of QDs modified GCEs and ECL sensor for IC50 determination

GCEs were firstly polished with 0.3 and 0.05 μm alumina slurry sequentially and sonicated in ethanol and distilled water for 3 min. The cleaned electrodes were dried with a steam of high-purity nitrogen gas. 10 μL of BPQDs, amino acid-functionalized BPQDs or peptide-functionalized BPQDs dispersion was then individually cast onto the GCEs and dried under ambient condition to get the QDs modified GCEs.

For ECL application, 10 µL dispersion of 5 mg mL^−1^ carboxylated MWNTs was dropped on the pretreated GCE and dried in a desiccator to get MWNTs/GCE. 20 µL of 0.1 M MES buffer containing 10 mM EDC and 5 mM NHS was then dropped onto MWNTs/GCE to incubate for 30 min at room temperature. After the activated MWNTs/GCE was thoroughly rinsed with 0.01 M pH 7.4 PBS, 20 µL of RRGDS-BPQDs was immediately dropped on its surface to incubate for 2 h at room temperature. The resulting RRGDS-BPQDs/MWNTs/GCE was rinsed with 0.01 M pH 7.4 PBS, and then was dropped with 20 µL of 1 × 10^6^ inhibitor-pretreated A549 cells mL^−1^ to incubate at 37 °C for 20 min. After carefully rinsing with 0.01 M pH 7.4 PBS, the obtained A549/RRGDS-BPQDs/MWNTs/GCE was used for subsequent assay. As control, MCF-7/RRGDS-BPQDs/MWNTs/GCE was prepared with the same procedure.

### ECL spectra

After 10 μL of BPQDs or R-BPQDs was dropped on GCE and dried, the cathodic and anodic ECL spectra of the modified electrodes were recorded in 0.1 M pH 7.4 PBS containing 0.1 M K_2_S_2_O_8_ or 25 mM N_2_H_4_∙H_2_O, respectively, with an optical filter from 380 to 700 nm in front of the PMT.

### Calculation method

All calculations were carried out using the Amsterdam Density Functional program package (ADF)^58^. The time-dependent density functional theory (TD-DFT) and DFT calculations were performed by applying the Perdew–Burke–Ernzerhof (PBE)^59^ exchange–correlation functional with the triple-zata plus polarization (TZP) basis set^60^. The ground state configuration, S_0_, of the oxidized BPQDs and R-BPQDs, was first optimized by DFT calculation. To simulate the emission, the configurations of the lowest singlet state, S_1_, and the lowest triplet state, T_1_, were relaxed using TD-DFT starting from the ground state configuration. We computed the vertical electronic transitions from the excited state relaxed configurations S_1_ and T_1_ to the ground-state, yielding the fluorescence and phosphorescence, respectively. The Van der Waals interaction was taken into account by the semi-empirical D3 method proposed by Grimme et al.^61^. All optimizations were done without any symmetry constraint.

### Reporting Summary

Further information on research design is available in the Nature Portfolio Reporting Summary linked to this article.

## Supplementary information

Supplementary Information

Reporting summary (printed in pdf)

## Data Availability

The data that support the findings of this study are available within the article and supplementary information files, or from the corresponding author upon request. Source data are provided with this paper.

## Source data

Source Data
