# Supplementary material for: Arginine-modified black phosphorus quantum dots with dual excited states for enhanced electrochemiluminescence in bioanalysis
Source: Nat Commun. 2022 Nov 26;13:7302. doi: 10.1038/s41467-022-35015-9 (PMC9701201; doi:10.1038/s41467-022-35015-9)
Supplement: Supplementary file 1 — Supplementary Information [file 41467_2022_35015_MOESM1_ESM.docx]

**Supplementary Information**

**Arginine-modified black phosphorus quantum dots with dual excited states for enhanced electrochemiluminescence in bioanalysis**

Siqi Yu^1,4^, Yu Du^1,4^, Xianghong Niu^2^, Guangming Li^1^, Da Zhu^1^, Qian Yu^1^, Guizheng Zou^3^ & Huangxian Ju^1^*

^1^State Key Laboratory of Analytical Chemistry for Life Science, School of Chemistry and Chemical Engineering, Nanjing University, Nanjing 210023, P. R. China. ^2^School of Science, Nanjing University of Posts and Telecommunications, Nanjing 210023, P. R. China. ^3^School of Chemistry and Chemical Engineering, Shandong University, Jinan 250100, P. R. China. ^4^These authors contributed equally. *e-mail: [hxju@nju.edu.cn](mailto:hxju@nju.edu.cn)

**Table of contents**

Supplementary Figures 1-19

Supplementary Tables 1-4

Supplementary Reference 1

Supplementary Figures


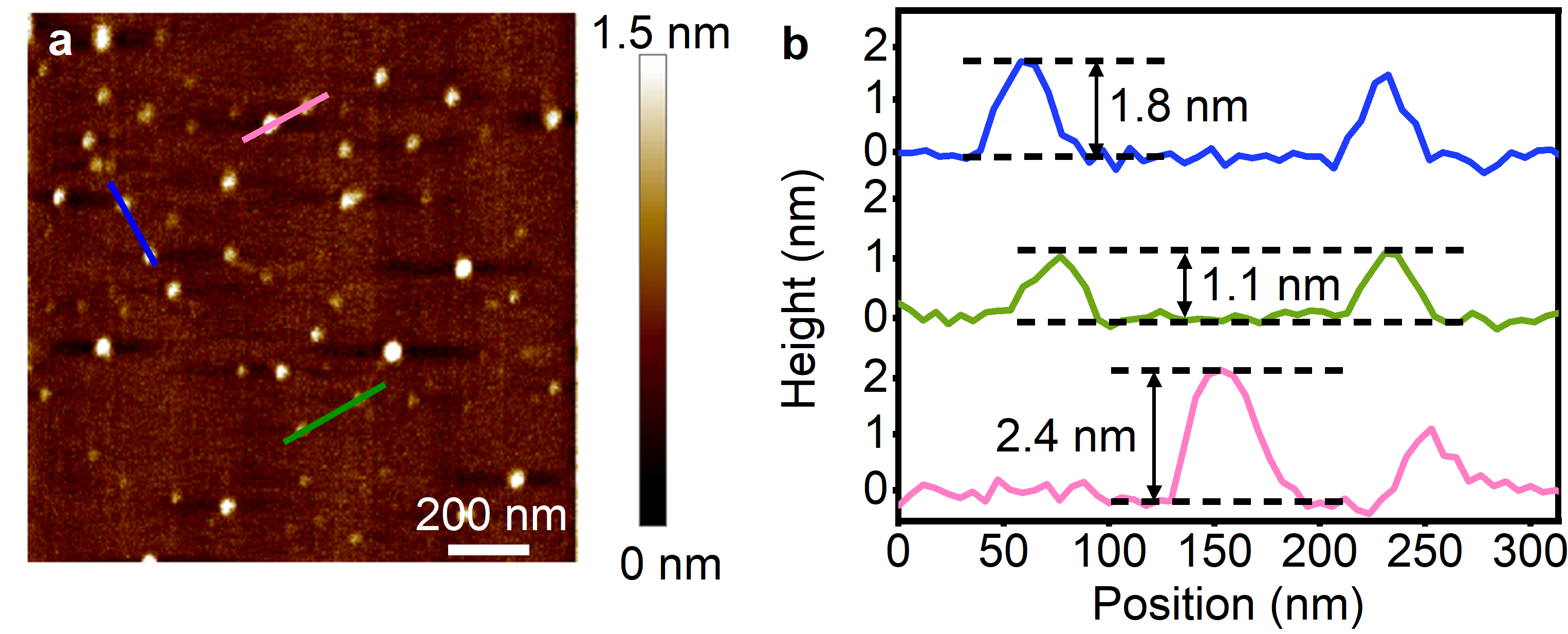


**Supplementary Figure 1 | AFM image and height profiles. a** AFM image of BPQDs. **b** Height profiles along the lines in (**a**).


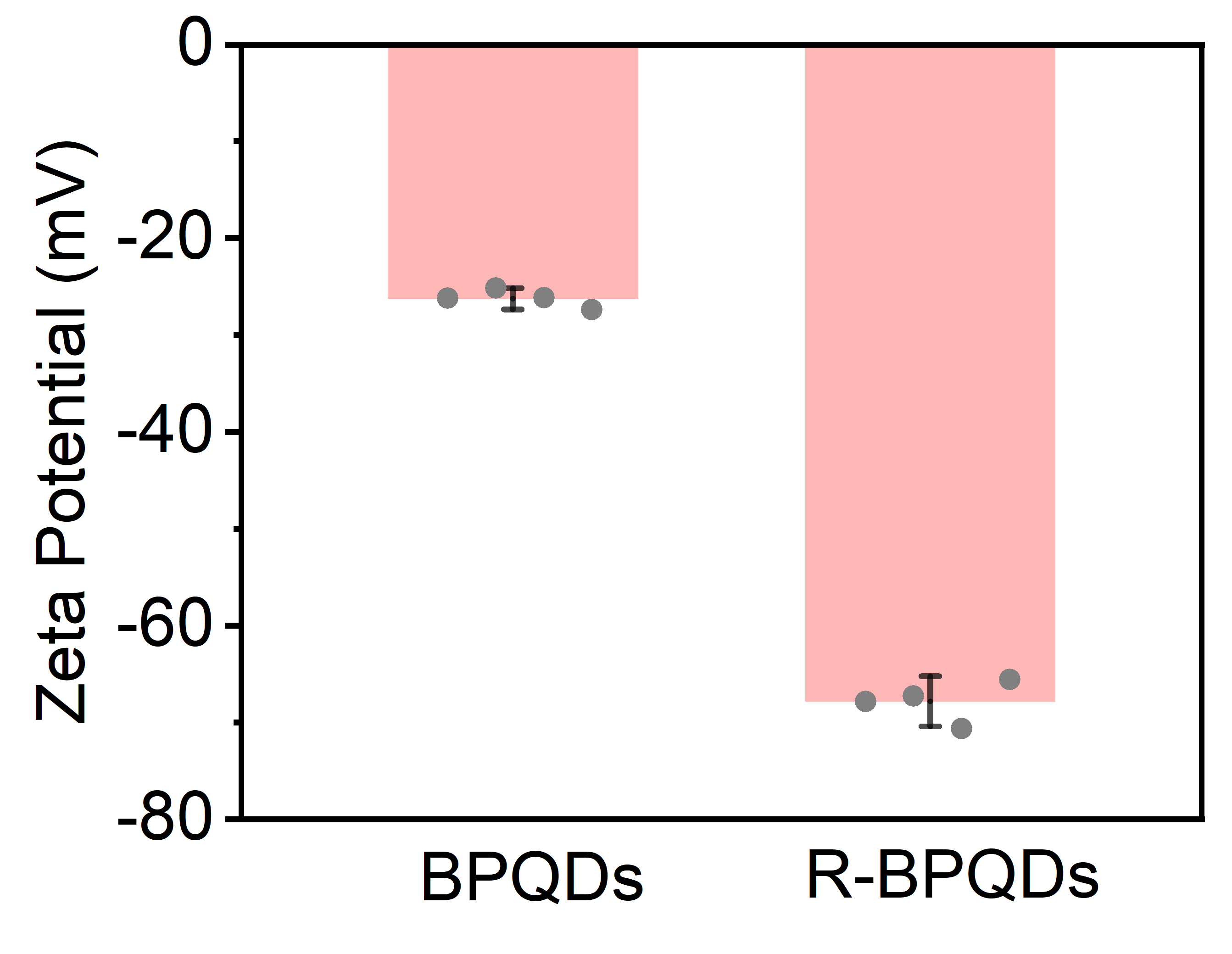


**Supplementary Figure 2 | Zeta potentials.** Zeta potentials of BPQDs and R-BPQDs. The error bars represent the SD from 4 measurements. Data are expressed as means ± SD.


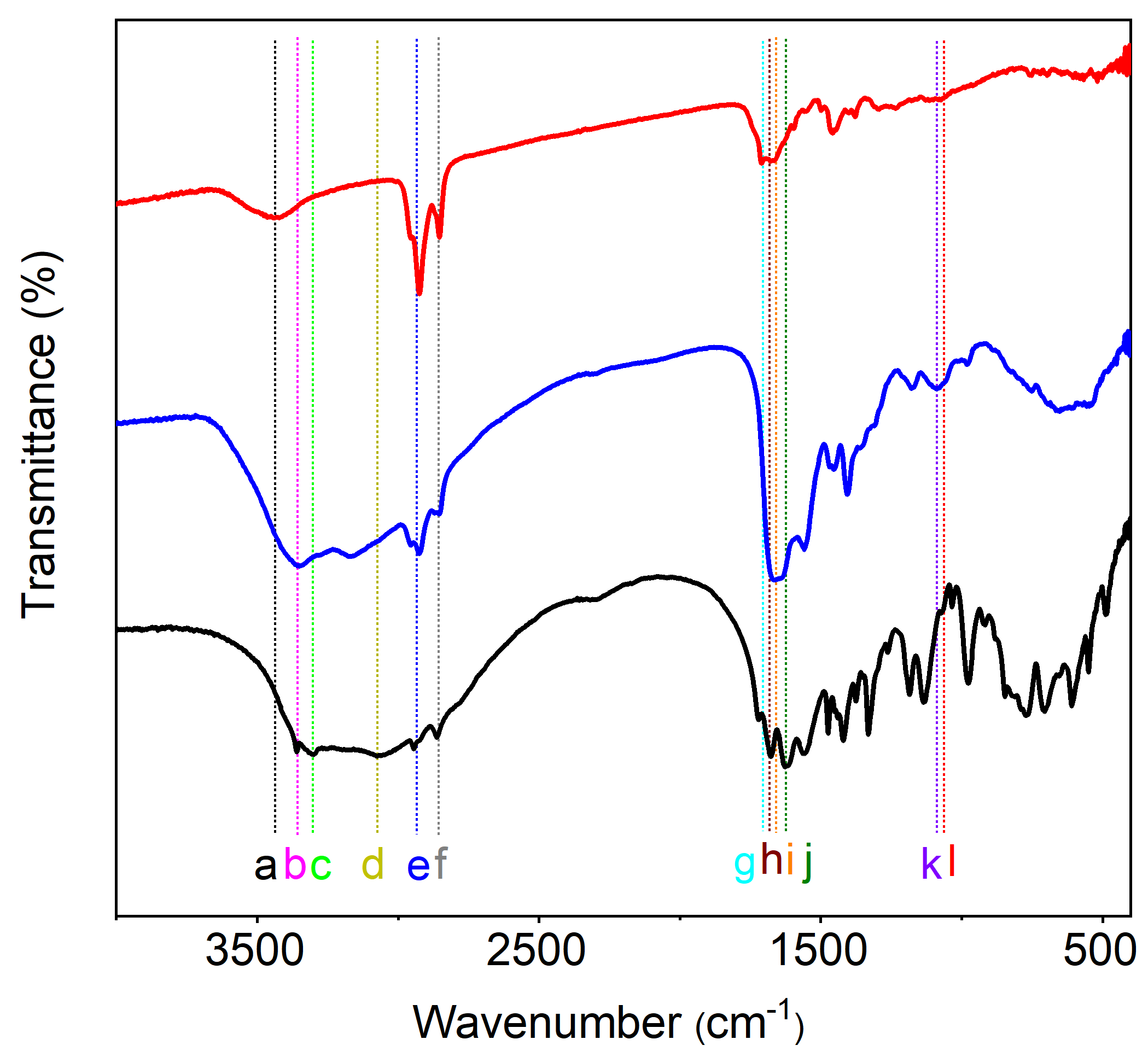


**Supplementary Figure 3 |** **Amplified FTIR spectra in Fig. 1c.** FTIR spectra of Arg (black line), R-BPQDs (blue line) and BPQDs (red line). Lines a to l represented the wavenumbers of 3473, 3410, 3346, 3115, 2973, 2895, 1730, 1721, 1705, 1678, 1089 and 1070 cm^-1^, respectively.


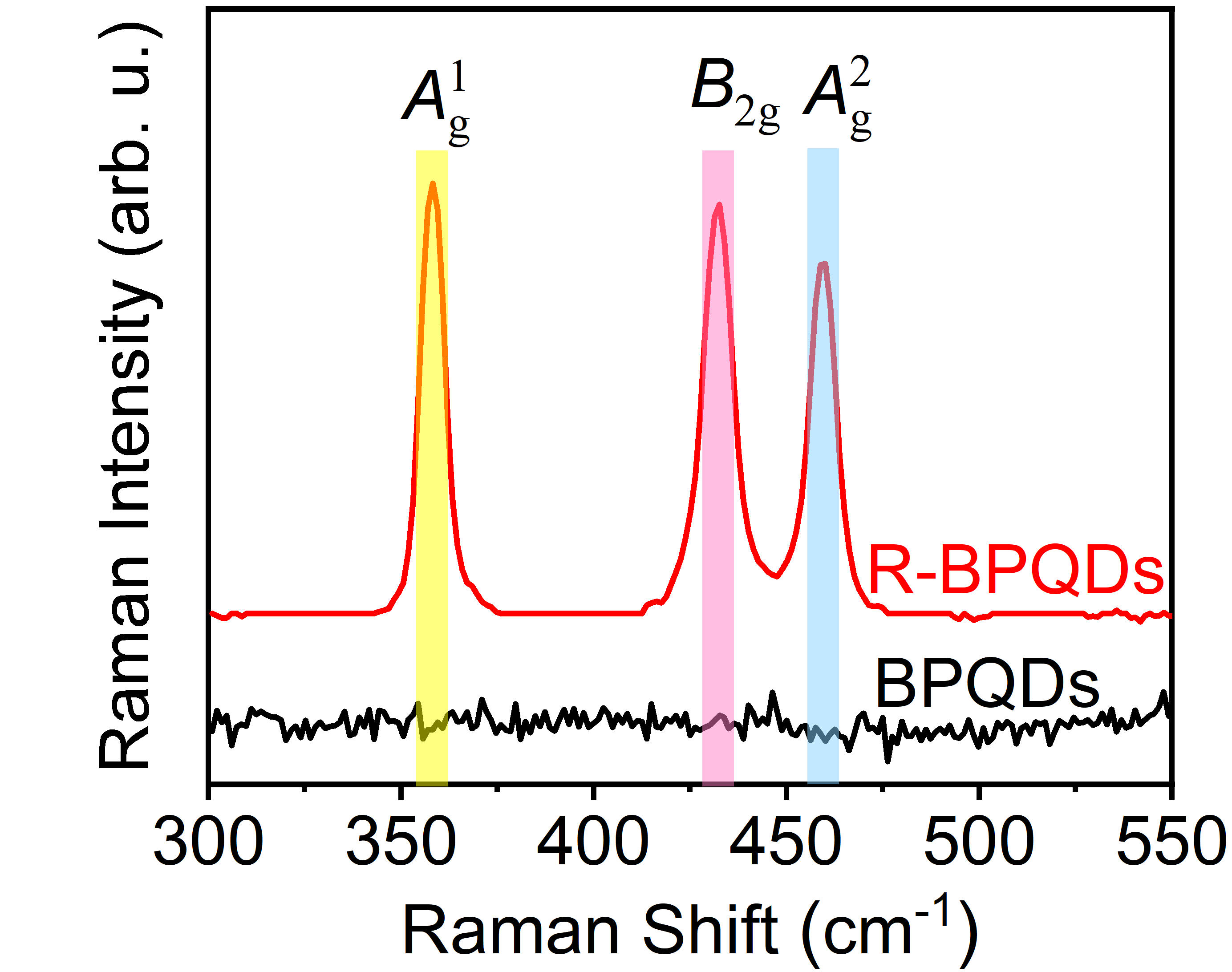


**Supplementary Figure 4 |** **Raman spectra of BPQDs and R-BPQDs.** Raman spectra of BPQDs and R-BPQDs after 30-day storage in dark under ambient conditions.


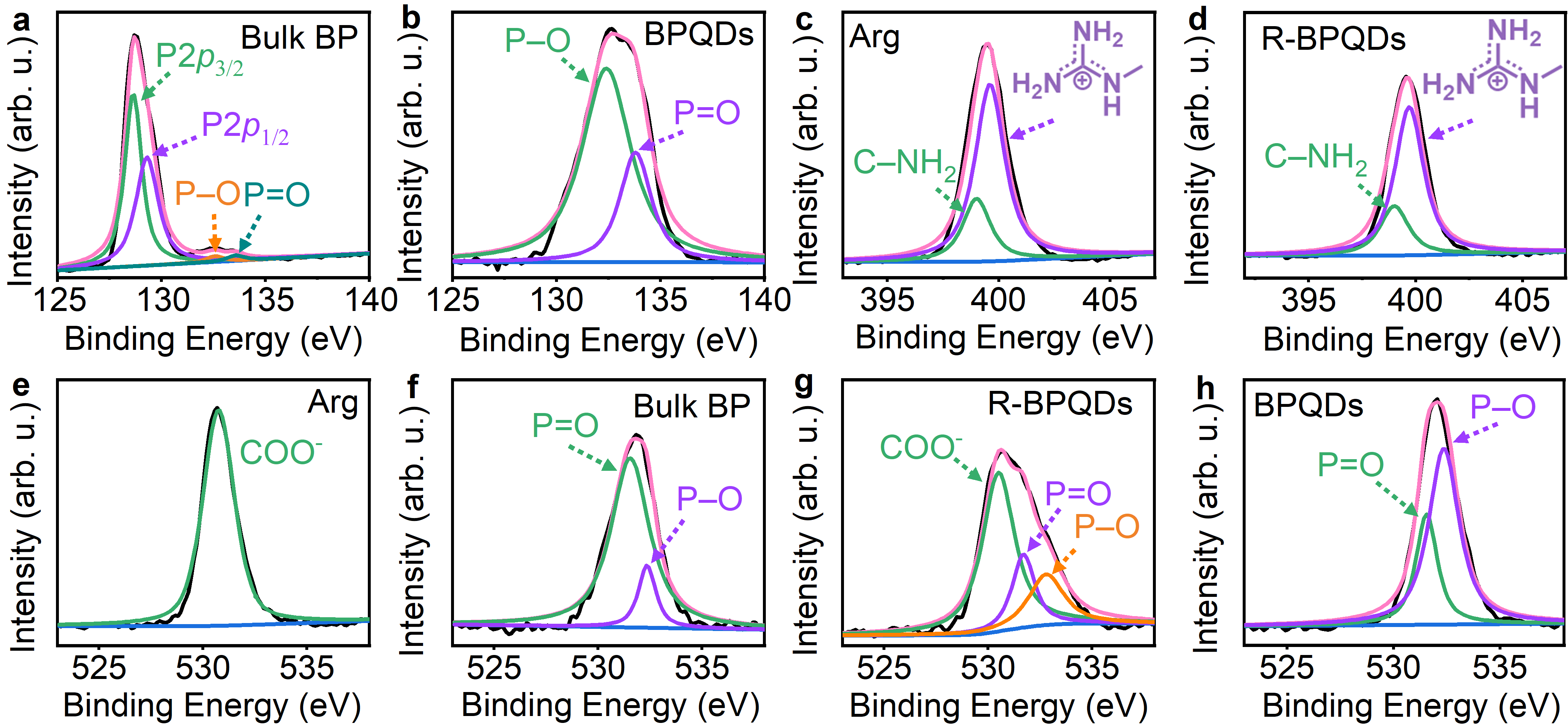


**Supplementary Figure 5 | XPS spectra.** P2*p* XPS spectra of **a** bulk BP and **b** BPQDs. N1*s* XPS spectra of **c** Arg and **d** R-BPQDs. O1*s* XPS spectra of **e** Arg, **f** bulk BP, **g** R-BPQDs and **h** BPQDs.


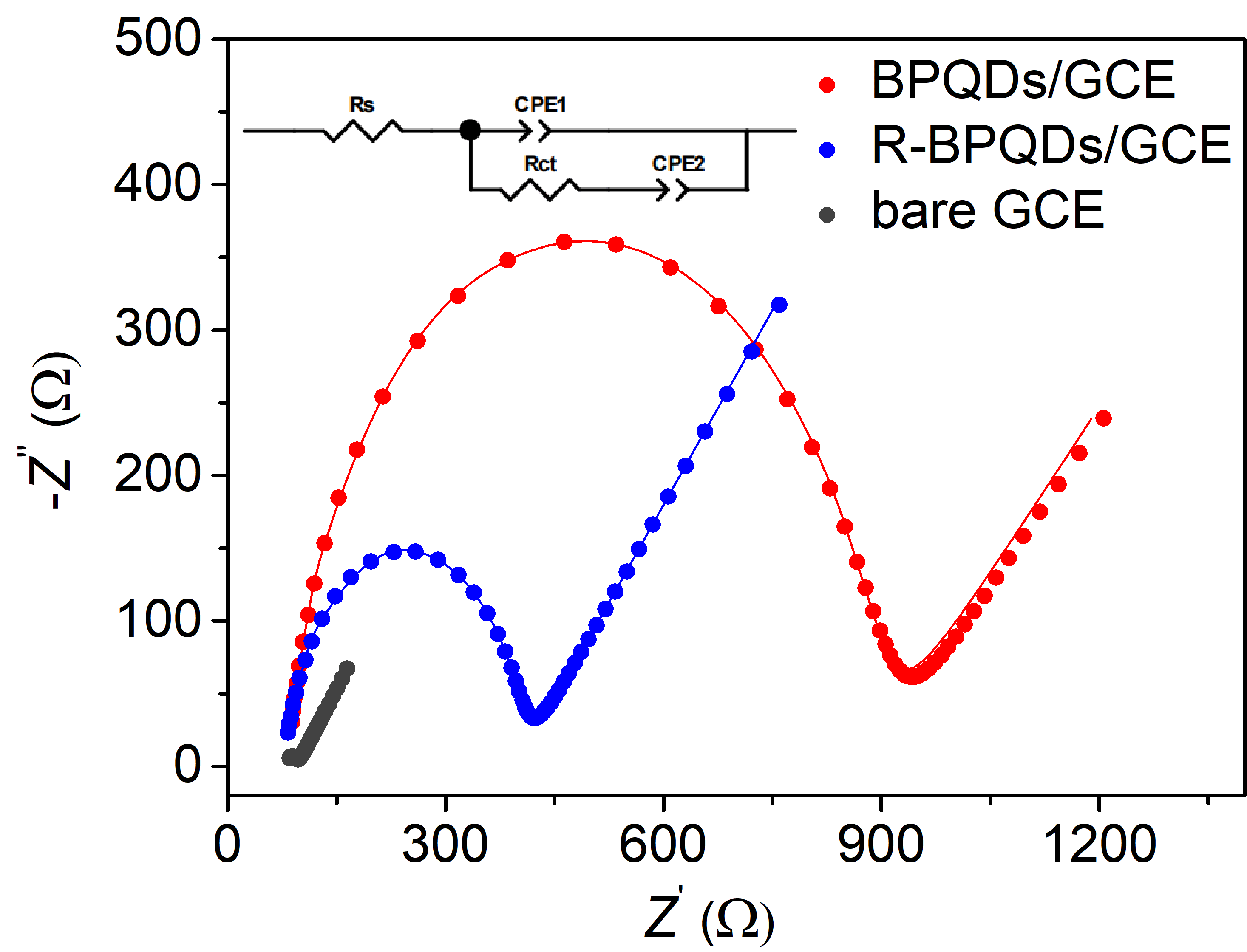


**Supplementary Figure 6 | Electrochemical impedance spectroscopic (EIS) measurements.** Nyquist plots (points) and simulations (lines) of bare and modified GCEs in 0.1 M KCl solution containing 5 mM K_4_[Fe(CN)_6_]/K_3_[Fe(CN)_6_] (1:1) in the frequency range from 0.01 Hz to 10 kHz with a signal amplitude of 10 mV. Inset: equivalent circuit.


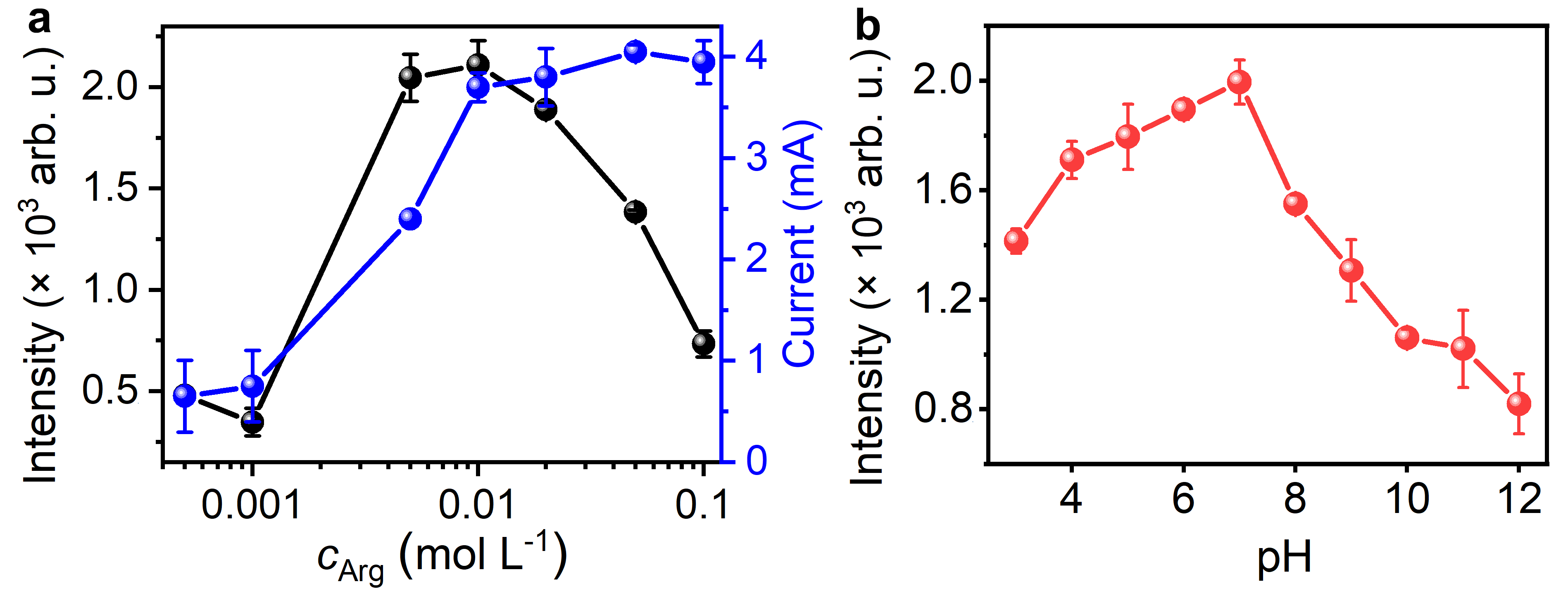


**Supplementary Figure 7 | ECL responses of different R-BPQDs.** Effects of **a** Arg concentration and **b** pH for preparation of R-BPQDs on ECL intensity or peak current of R-BPQDs/GCE. PMT = 600 V. The error bars represent the SD from 3 measurements. Data are expressed as means ± SD.


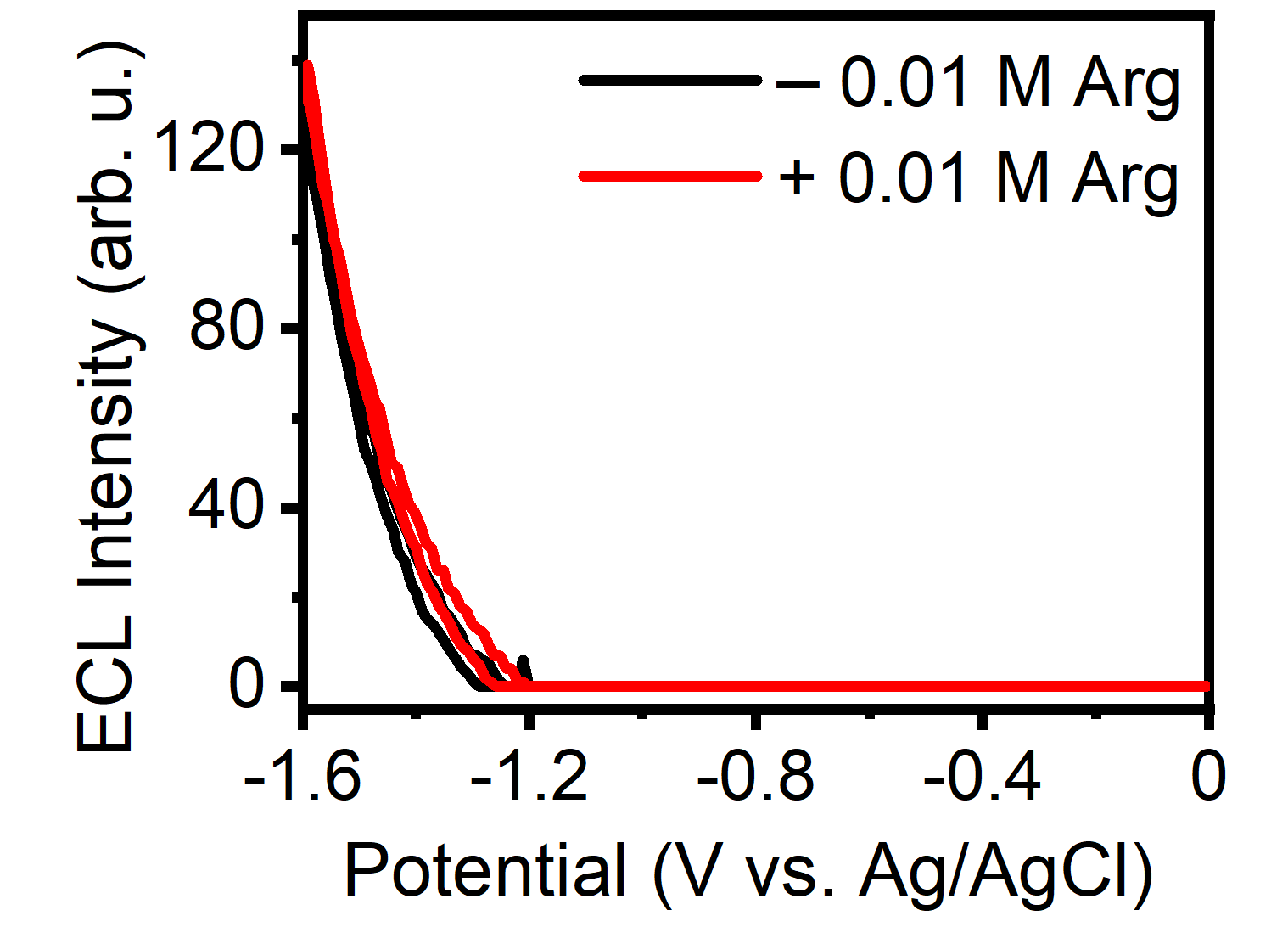


**Supplementary Figure 8 | Cathodic ECL curves of Arg.** Cathodic ECL curves of GCE in 0.1 M PBS containing 0.1 M K_2_S_2_O_8_ in absence or presence of 0.01 M Arg. PMT = 600 V.


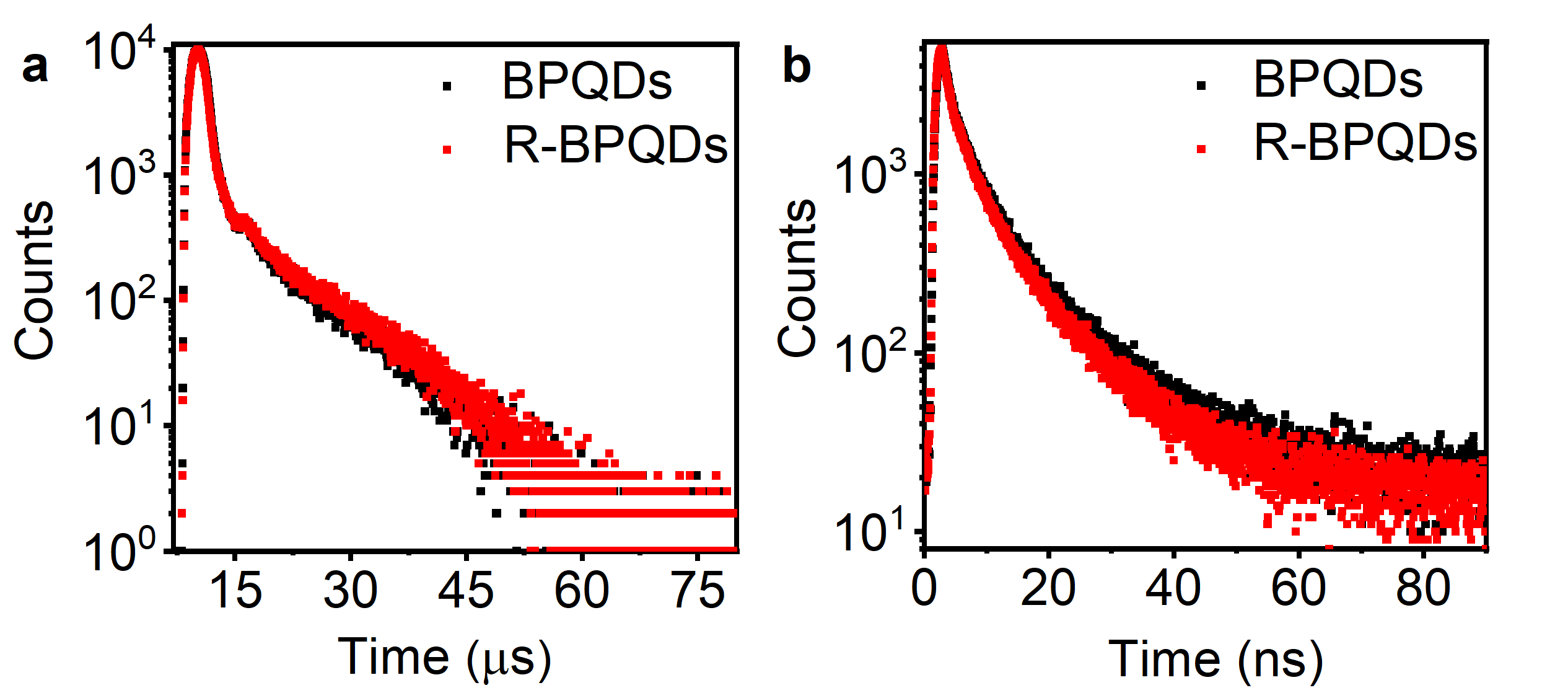


**Supplementary Figure 9 | Photoluminescence decay spectra.** Photoluminescence decay spectra of **a** BPQDs and R-BPQDs dispersed in water at *λ*_ex_ of 500 nm and *λ*_em_ of 580 nm, and **b** BPQDs at *λ*_ex_ of 405 nm and *λ*_em_ of 520 nm and R-BPQDs at *λ*_ex_ of 365 nm and *λ*_em_ of 460 nm.


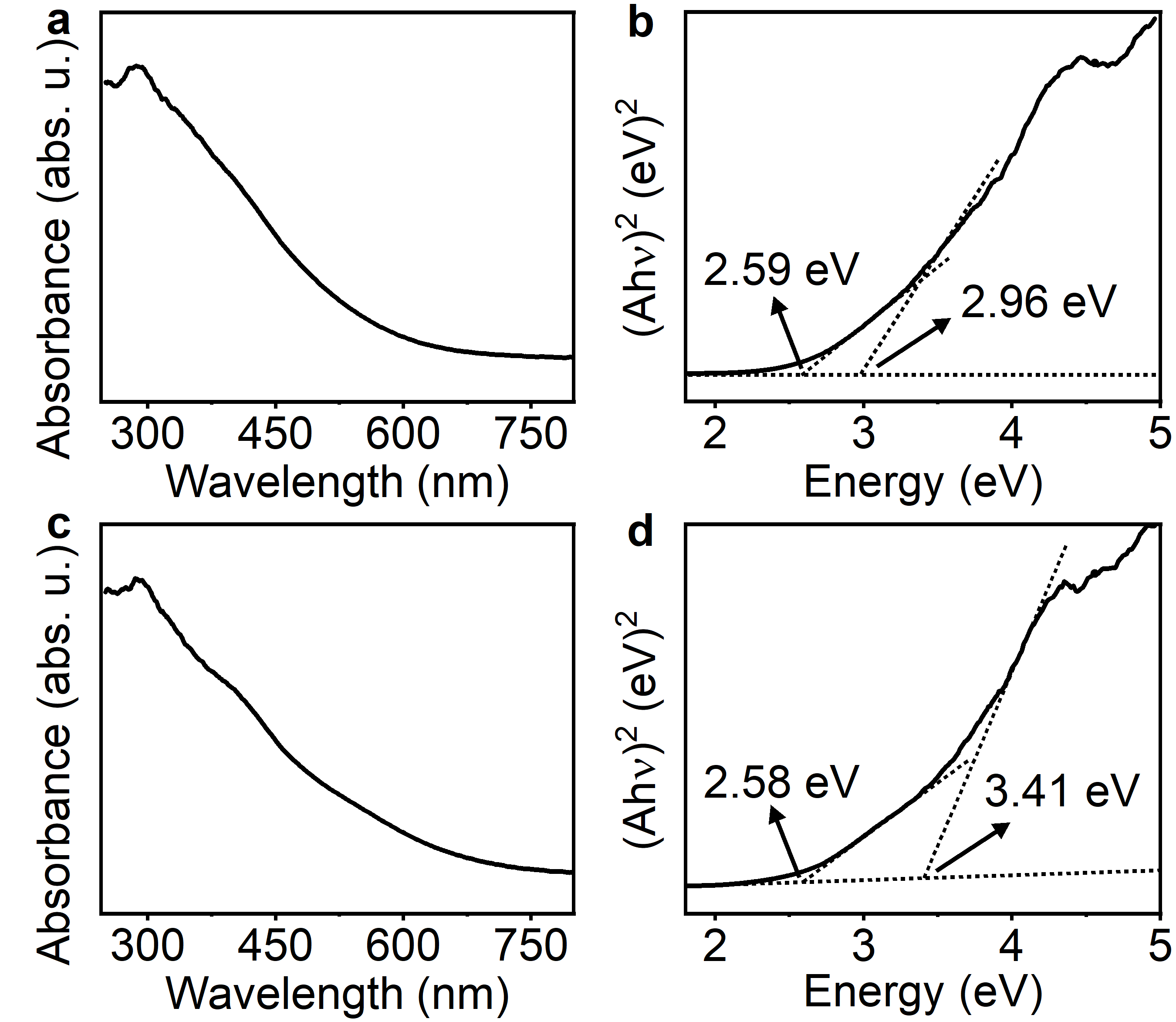


**Supplementary Figure 10 | UV-vis-NIR diffuse reflectance spectra. a**, **c** UV-vis-NIR diffuse reflectance spectra and **b**, **d** corresponding Tauc plots of BPQDs (**a**, **b**) and R-BPQDs (**c**, **d**).


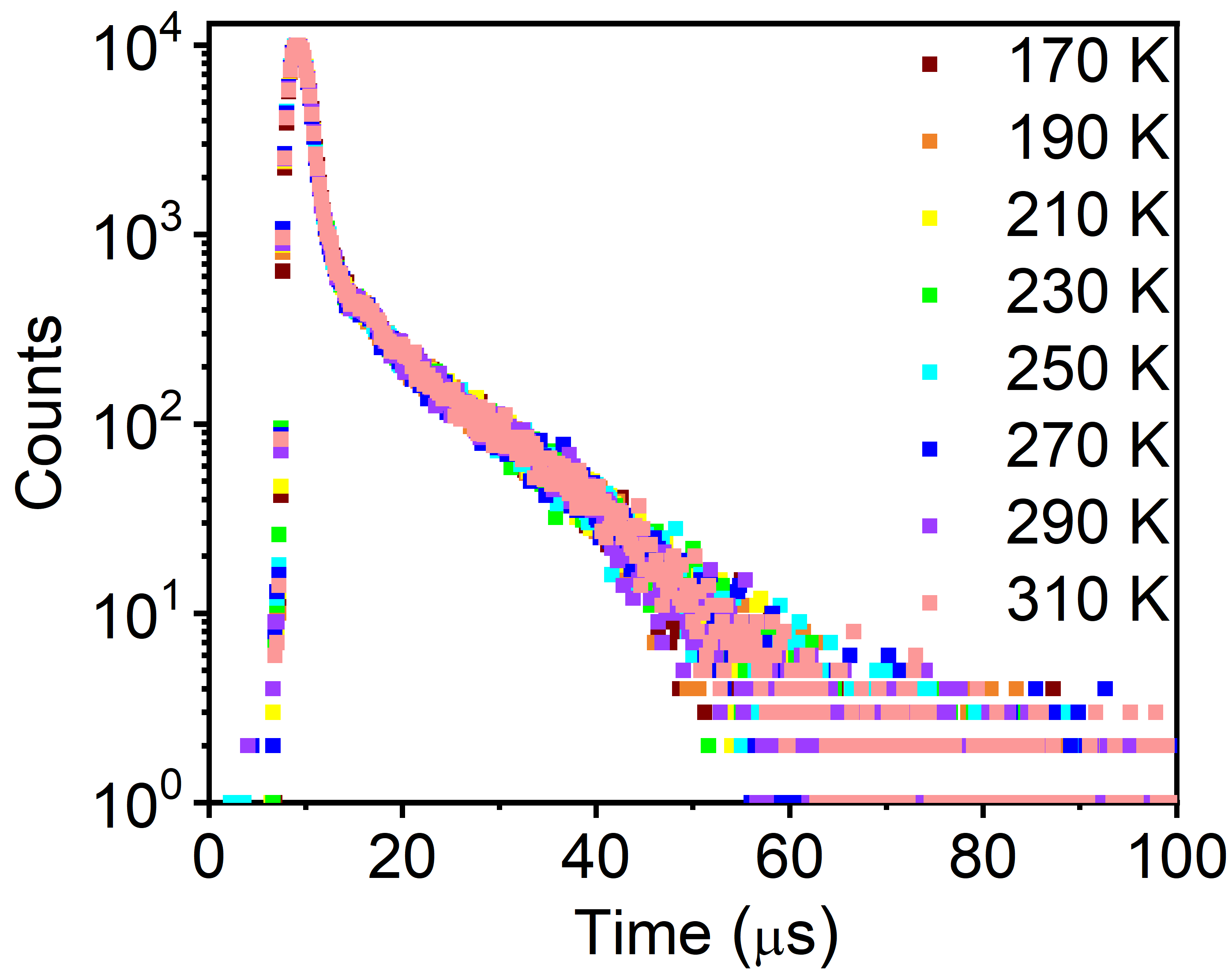


**Supplementary Figure 11 | Photoluminescence decay spectra** **in n-propanol.** Photoluminescence decay spectra of R-BPQDs in n-propanol at marked temperatures (*λ*_ex_ = 500 nm, *λ*_em_ = 580 nm).





**Supplementary Figure 12 | CV curve of Arg.** CV curve of 0.1 M PBS (pH 7.4) containing 0.01 M Arg at 0.1 V s^-1^.


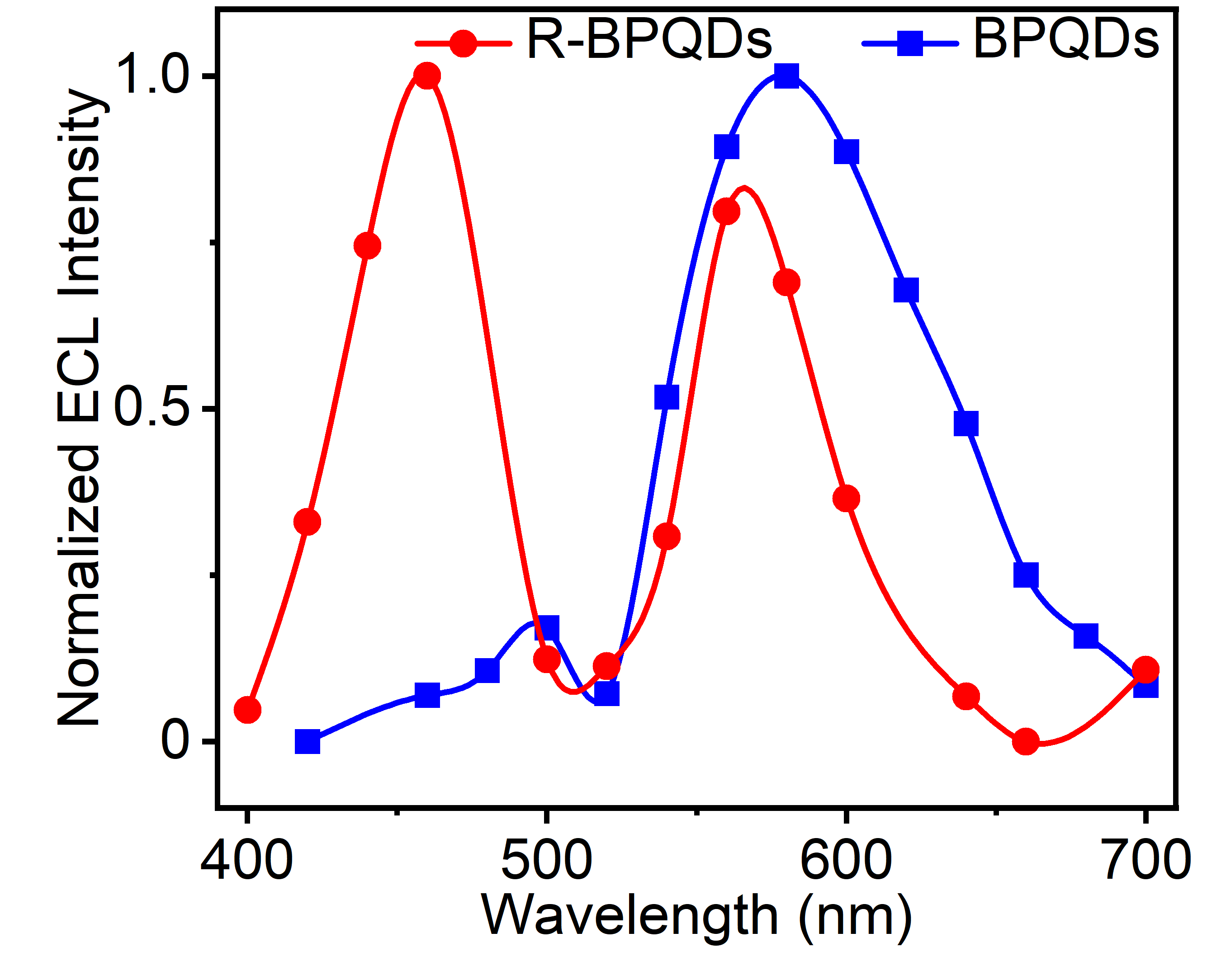


**Supplementary Figure 13 | Anodic ECL spectra.** Normalized anodic ECL spectra of BPQDs/GCE and R-BPQDs/GCE in 0.1 M PBS containing 25 mM N_2_H_4_∙H_2_O. PMT = 800 V.


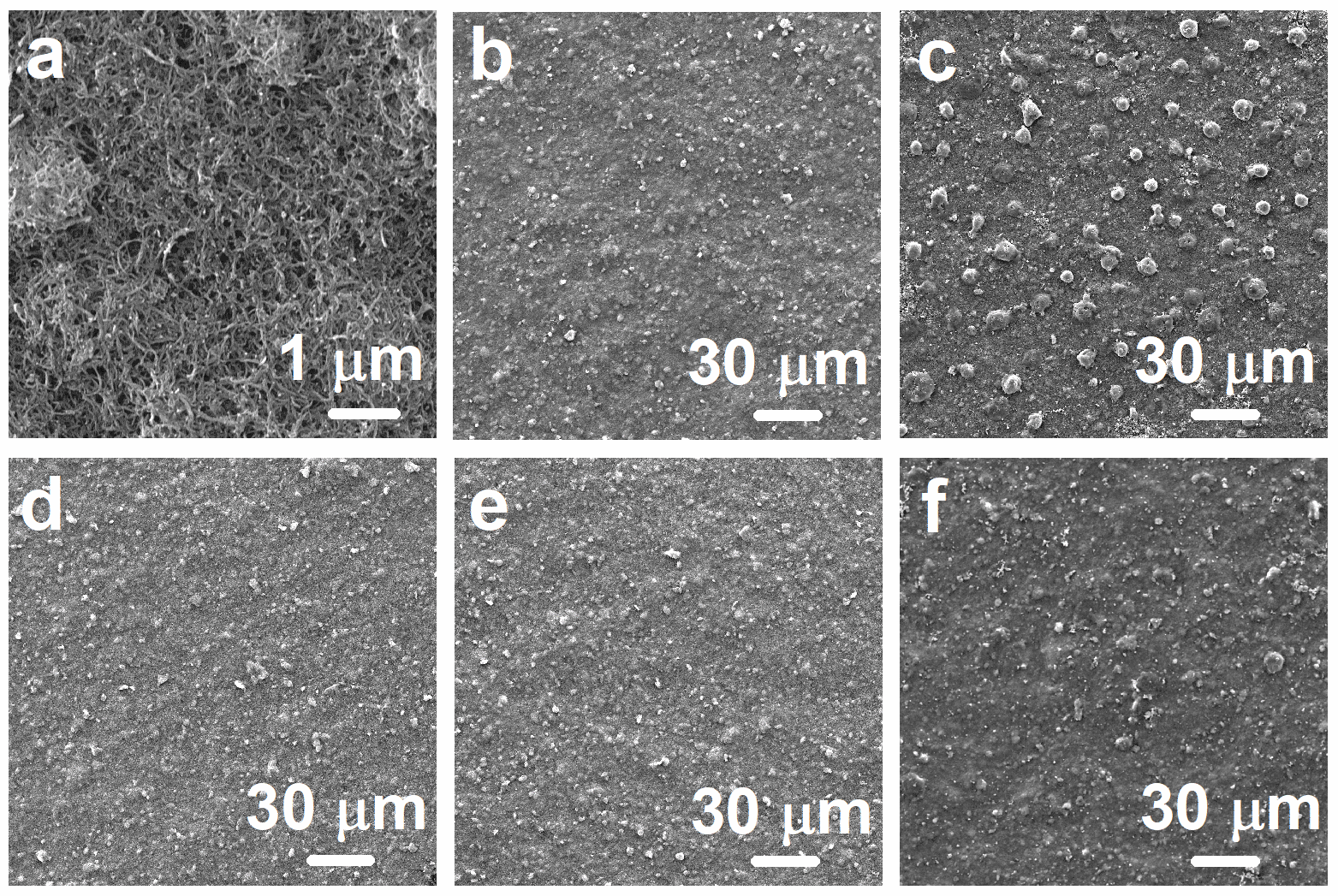


**Supplementary Figure 14 | SEM images of different MWNTs/FTO.** SEM images of **a** and **d** MWNTs/FTO, **b** RRGDS-BPQDs/MWNTs/FTO, **c** A549/RRGDS-BPQDs/MWNTs/FTO, **e** GGGDS-BPQDs/MWNTs/FTO and **f** A549/GGGDS-BPQDs/MWNTs/FTO.


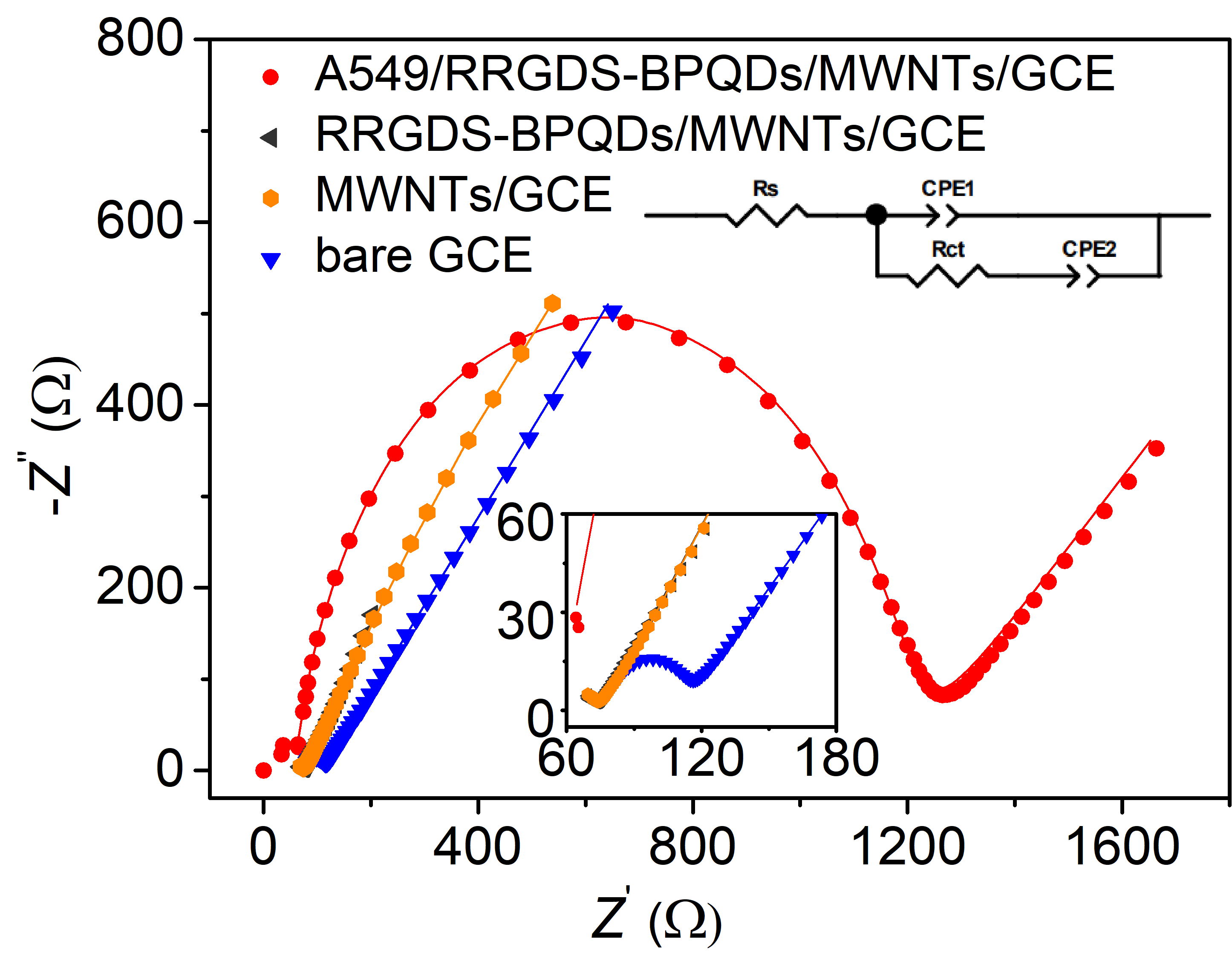


**Supplementary Figure 15 | EIS Nyquist plots of different MWNTs/GCEs.** EIS Nyquist plots (points) and simulations (lines) of GCE, MWNTs/GCE, RRGDS-BPQDs/MWNTs/GCE and A549/ RRGDS-BPQDs/MWNTs/GCE in 0.10 M KCl containing 5 mM K_4_[Fe(CN)_6_]/K_3_[Fe(CN)_6_] (1:1) in the frequency range from 0.01 Hz to 10 kHz with a signal amplitude of 10 mV. Inset: equivalent circuit.


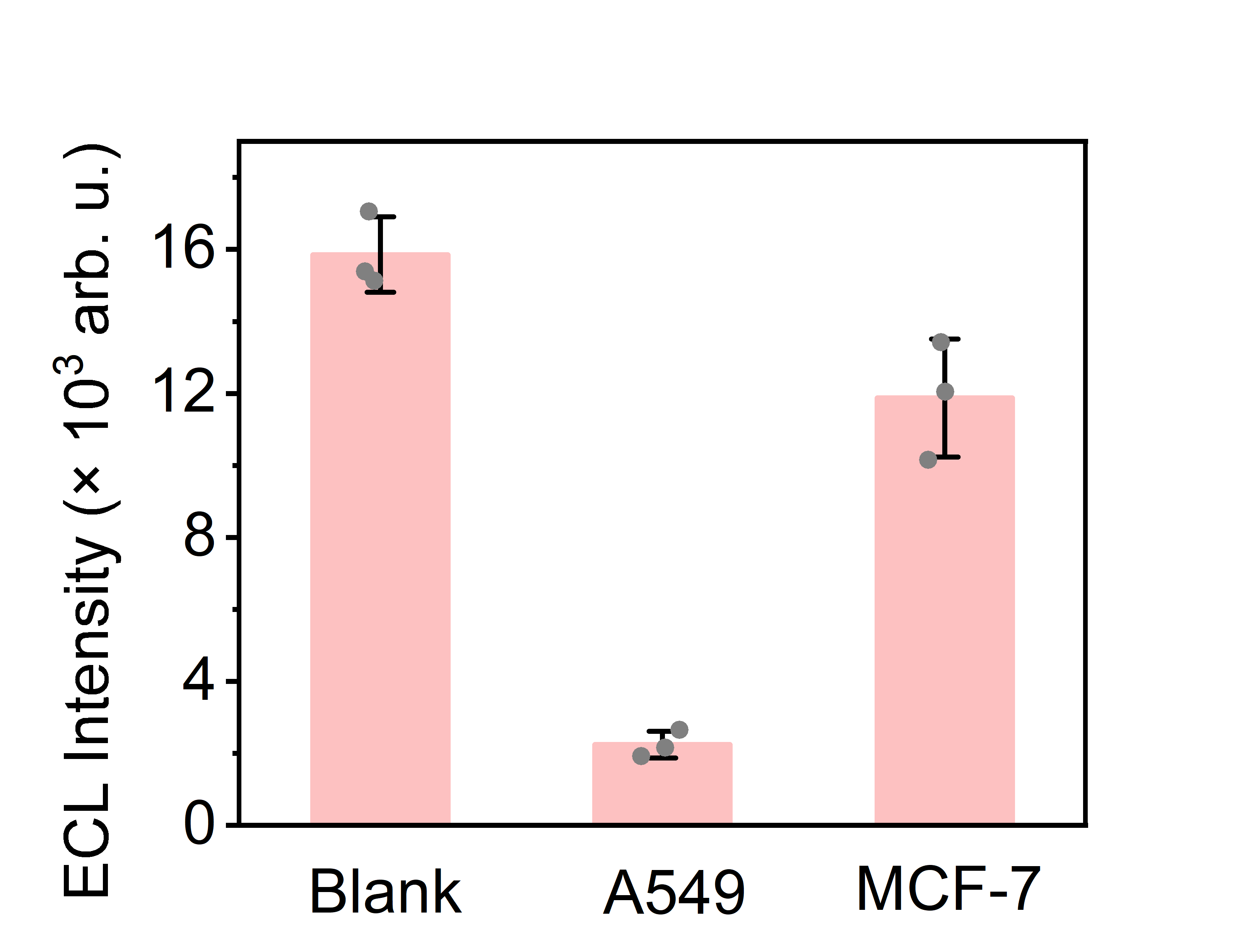


**Supplementary Figure 16 | ECL responses of different MWNTs/GCEs.** ECL intensities of RRGDS-BPQDs/MWNTs/GCE (blank), A549/RRGDS-BPQDs/MWNTs/GCE and MCF-7/RRGDS-BPQDs/MWNTs/GCE in 0.1 M PBS containing 0.1 M K_2_S_2_O_8_. PMT = 800 V. The error bars represent the SD from 3 measurements. Data are expressed as means ± SD.


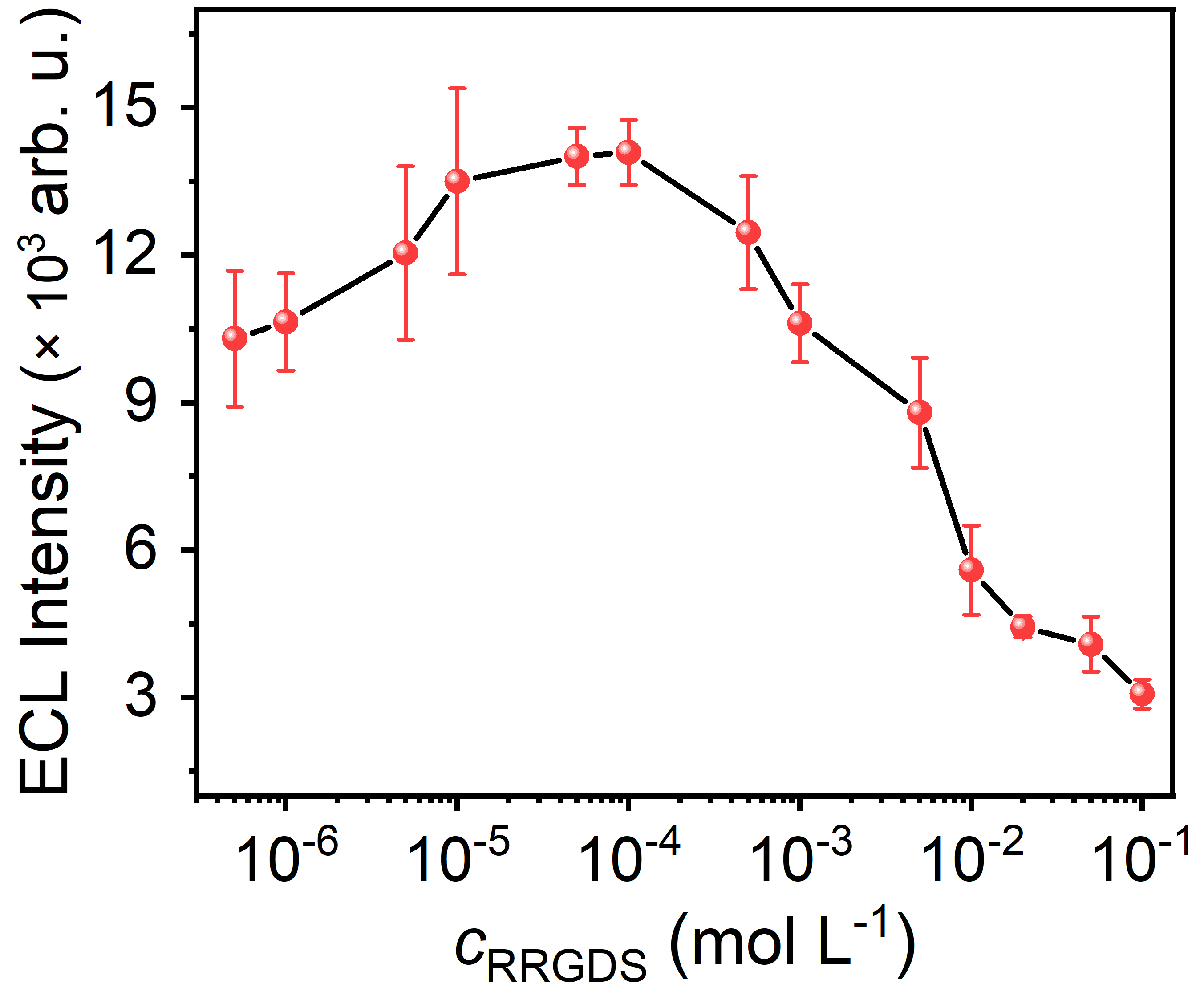


**Supplementary Figure 17 | ECL intensity of RRGDS-BPQDs/GCEs.** Effect of RRGDS peptide concentration for preparation of RRGDS-BPQDs on ECL intensity of RRGDS-BPQDs/GCE in 0.1 M PBS containing 0.1 M K_2_S_2_O_8_. PMT = 800 V. The error bars represent the SD from 3 measurements. Data are expressed as means ± SD.


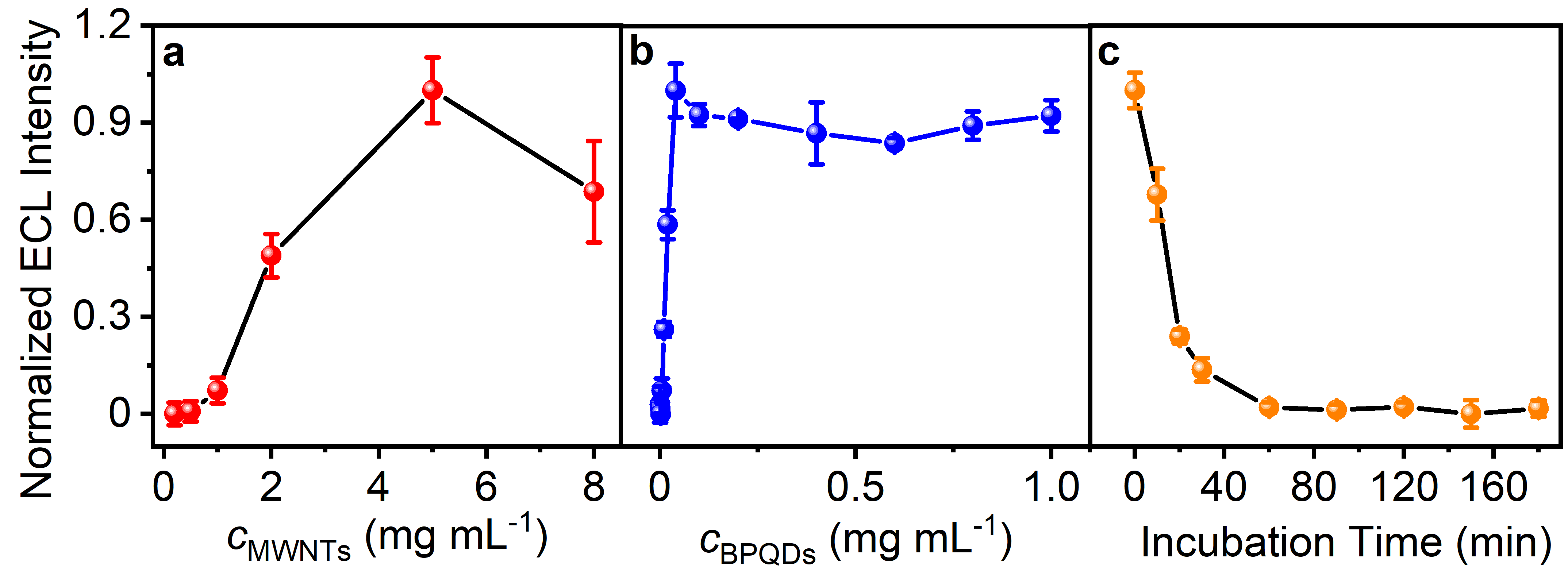


**Supplementary Figure 18 | Condition optimization for evaluation of integrin inhibitor.** Effects of **a** MWNTs concentration, **b** BPQDs concentration and **c** incubation time of A549 cells for preparation of A549/RRGDS-BPQDs/MWNTs/GCE on ECL intensity in 0.1 M PBS containing 0.1 M K_2_S_2_O_8_. PMT = 800 V. The error bars represent the SD from 3 measurements. Data are expressed as means ± SD.


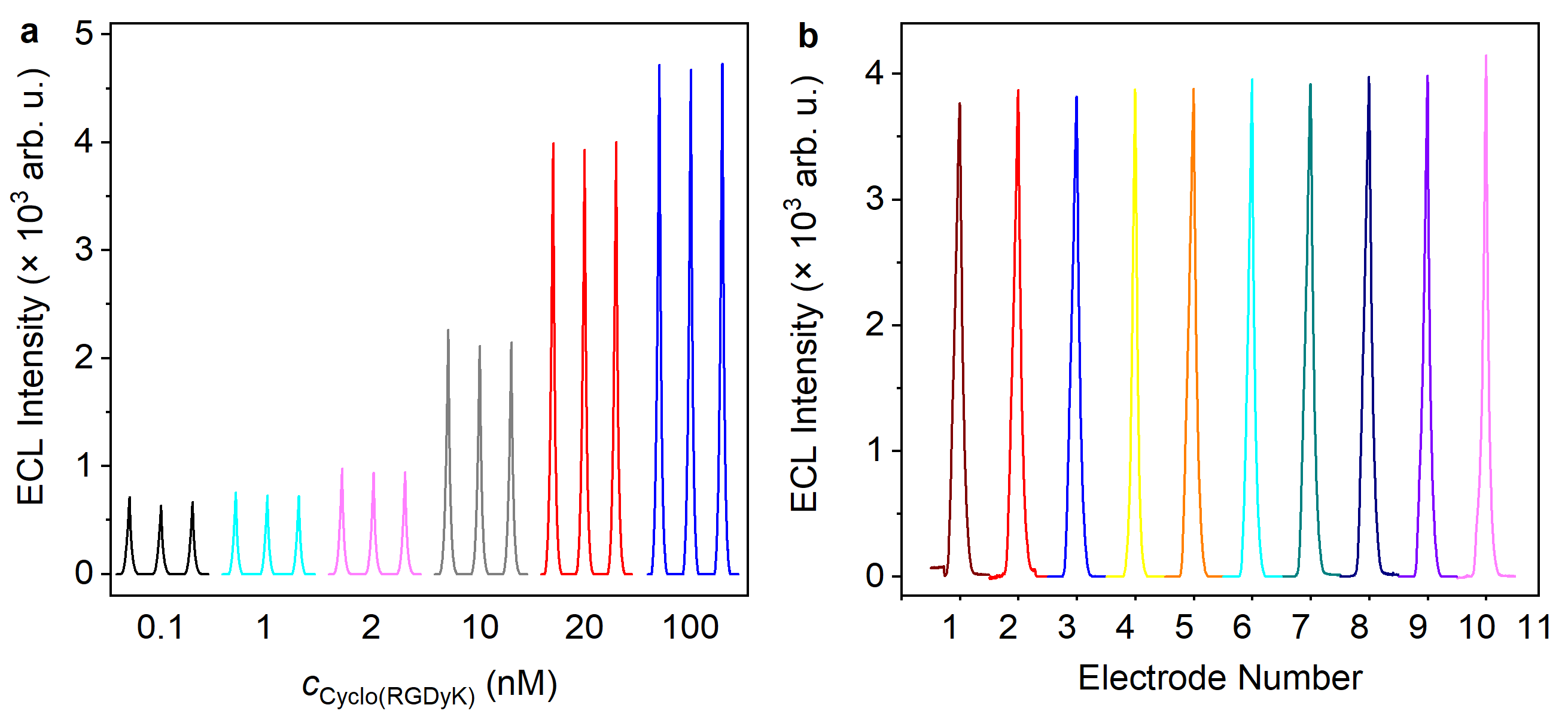


**Supplementary Figure 19 | Stability and reproducibility for evaluation of integrin inhibitor. a** Stability of the ECL sensor for continous measurments at different concentrations of cyclo(RGDyK). **b** Reproducibility of ten A549/RRGDS-BPQDs/MWNTs/GCEs with 20 nM cyclo(RGDyK). PMT = 800 V.

**Supplementary Tables**

**Supplementary Table 1 |** Assignments of FTIR peaks for Arg, BPQDs and R-BPQDs in Supplementary Fig. 3.

| Sample | Peak (cm^-1^) | Assignment |
| --- | --- | --- |
| Arg | 1678 | COO^-^ asymmetric stretching |
|  | 1721 | N−H bending vibration |
|  | 2895, 2973, 3115 | C−H stretching vibration |
|  | 3346, 3410 | N−H stretching vibration |
| BPQDs | 1070 | P−O bending vibration |
|  | 1730 | P=O vibration |
|  | 2895, 2973 | P−O vibration |
|  | 3473 | O−H vibration |
| R-BPQDs | 1089 | P−O bending vibration |
|  | 1705 | N−H bending vibration |
|  | 2895, 2973 | P−O vibration |
|  | 2895, 2973, 3115 | C−H stretching vibration |
|  | 3346, 3410 | N−H stretching vibration |
|  | 3473 | O−H vibration |

**Supplementary Table 2 |** Fitting parameters of photoluminescence decay curves for BPQDs and R-BPQDs in water at corresponding PL emission region (*λ*_ex_ = 500 nm, *λ*_em_ = 580 nm). The average lifetimes (*τ*_avg_) were calculated according to equation (1),^[1]^ where *τ_i_* and *B_i_* (*i* = 1, 2) are lifetime and weight factor for each lifetime component, respectively. *χ*^2^ represents the confidence factor.

| Sample | *τ*_1_ (μs) | *B*_1_ (%) | *τ*_2_ (μs) | *B*_2_ (%) | *τ*_avg_ (μs) | *χ*^2^ |
| --- | --- | --- | --- | --- | --- | --- |
| BPQDs | 3.080 | 15.61 | 12.27 | 84.39 | 11.86 | 1.030 |
| R-BPQDs | 1.821 | 10.92 | 13.14 | 89.08 | 12.95 | 1.127 |

$$\tau_{\mathrm{avg}}=\frac{\sum B_{i}\tau_{i}^{2}}{\sum B_{i}\tau_{i}}$$

(1)

**Supplementary Table 3 |** Fitting parameters of photoluminescence decay curves for BPQDs (*λ*_ex_ = 405 nm, *λ*_em_ = 520 nm) and R-BPQDs (*λ*_ex_ = 365 nm, *λ*_em_ = 460 nm) in water at corresponding FL emission region.

| Sample | *τ*_1_ (ns) | *B*_1_ (%) | *τ*_2_ (ns) | *B*_2_ (%) | *τ*_3_ (ns) | *B*_3_ (%) | *τ*_avg_ (ns) | *χ*^2^ |
| --- | --- | --- | --- | --- | --- | --- | --- | --- |
| BPQDs | 0.8102 | 7.570 | 4.062 | 48.77 | 13.06 | 43.66 | 10.66 | 1.128 |
| R-BPQDs | 0.7316 | 6.630 | 3.644 | 47.04 | 11.35 | 46.33 | 9.396 | 1.136 |

**Supplementary Table 4 |** Fitting parameters of photoluminescence decay curves for R-BPQDs in n-propanol at temperatures from 170 to 310 K (*λ*_ex_ = 500 nm, *λ*_em_ = 580 nm).

| Temperature (K) | *τ*_1_ (μs) | *B*_1_ (%) | *τ*_2_ (μs) | *B*_2_ (%) | *τ*_avg_ (μs) | *χ*^2^ |
| --- | --- | --- | --- | --- | --- | --- |
| 170 | 1.061 | 0.1122 | 9.402 | 0.8878 | 8.466 | 1.124 |
| 190 | 1.154 | 0.1219 | 9.866 | 0.8781 | 8.804 | 1.306 |
| 210 | 0.8930 | 0.1139 | 9.693 | 0.8861 | 8.691 | 1.119 |
| 230 | 0.9987 | 0.1187 | 9.690 | 0.8813 | 8.658 | 1.222 |
| 250 | 0.9272 | 0.1211 | 9.814 | 0.8789 | 8.738 | 1.184 |
| 270 | 1.064 | 0.1138 | 9.746 | 0.8862 | 8.758 | 1.333 |
| 290 | 0.8830 | 0.1103 | 9.408 | 0.8897 | 8.468 | 1.325 |
| 310 | 1.041 | 0.1199 | 9.795 | 0.8801 | 8.745 | 1.137 |

**Supplementary Reference**

1. Li, W. et al. A universal strategy for activating the multi-color room-temperature afterglow of carbon dots in a boric acid matrix. *Angew. Chem. Int. Ed.* **131**, 7356–7361 (2019).
